# Parallel or convergent evolution in human population genomic data revealed by genotype networks

**DOI:** 10.1186/s12862-016-0722-0

**Published:** 2016-08-02

**Authors:** Ali R. Vahdati, Andreas Wagner

**Affiliations:** 1 Institute of Evolutionary Biology and Environmental Studies, University of Zurich, Zurich, Switzerland; 2 The Swiss Institute of Bioinformatics, Lausanne, Switzerland; 3 The Santa Fe Institute, Santa Fe, USA

**Keywords:** Genotype networks, Genetic variation, Natural selection, Human genome

## Abstract

**Background:**

Genotype networks are representations of genetic variation data that are complementary to phylogenetic trees. A genotype network is a graph whose nodes are genotypes (DNA sequences) with the same broadly defined phenotype. Two nodes are connected if they differ in some minimal way, e.g., in a single nucleotide.

**Results:**

We analyze human genome variation data from the 1,000 genomes project, and construct haploid genotype (haplotype) networks for 12,235 protein coding genes. The structure of these networks varies widely among genes, indicating different patterns of variation despite a shared evolutionary history. We focus on those genes whose genotype networks show many cycles, which can indicate homoplasy, i.e., parallel or convergent evolution, on the sequence level.

**Conclusion:**

For 42 genes, the observed number of cycles is so large that it cannot be explained by either chance homoplasy or recombination. When analyzing possible explanations, we discovered evidence for positive selection in 21 of these genes and, in addition, a potential role for constrained variation and purifying selection. Balancing selection plays at most a small role. The 42 genes with excess cycles are enriched in functions related to immunity and response to pathogens. Genotype networks are representations of genetic variation data that can help understand unusual patterns of genomic variation.

**Electronic supplementary material:**

The online version of this article (doi:10.1186/s12862-016-0722-0) contains supplementary material, which is available to authorized users.

## Background

The patterns and causes of genotypic variation in human genes have been a focus of great recent interest in evolutionary biology. Different processes such as natural selection, genetic recombination, genetic drift, demography, as well as physicochemical properties of cells, can influence this diversity. Various methods have been devised to represent and quantify genetic variation and to detect its causes [1–10].

Here we use a novel approach based on genotype networks to represent and analyze genetic variation in human genes. Genotype networks are graphs that consist of nodes, which correspond to genotypes with the same phenotype, where sameness can be defined as narrowly as enzyme activity, or as broadly as viability. Nodes that differ in some minimal way from each other are adjacent, i.e., connected by an edge in such a graph. The genotypes we consider are haploid genotypes (haplotypes) of human genes in a sample of the human population, and we call two genotypes adjacent if they differ in a single nucleotide. Genotype networks can be useful to address various evolutionary questions, such as how novel adaptations originate, and what role phenotypic robustness or plasticity play in adaptation [11]. In the past, they have been mostly built from computational models of genotype-phenotype maps [12–15], but high-throughput genotyping allows us to build genotype networks from experimental data [16]. Representing such data in the form of a network makes the large analytical toolbox of graph theory available, which has been useful in fields as different as ecology and the social sciences [17–20].

A common form of representation for genetic variation data is the phylogenetic tree, which shows the evolutionary relationship among a set of taxa, individuals, or genes that constitute the leaves of the tree. The common ancestors of these taxa form the interior nodes of such a tree. In a gene tree, these ancestors can be reconstructed with the help of probabilistic models of sequence evolution [21–23]. Phylogenetic trees are by definition *acyclic* graphs: They do not contain cycles – paths of edges that start from a node, pass through other nodes, and return to the same node.

The acyclic nature of phylogenetic trees implies one major limitation of such trees: They cannot easily accommodate evolutionary genealogies more complex than simple vertical descent with modification [24–27]. Such genealogies can lead to reticulate networks of phylogenetic relationships. Thus, multiple mechanisms to create genetic diversity, such as hybridization, allopolyploidization, sexual reproduction, recombination, gene conversion, and homoplasy, which lead to mosaic patterns of relationships among nodes are not easily accommodated in tree-like structures. Genotype networks provide information complementary to phylogenetic trees that are not subject to this limitation, because they can accommodate cycles.

**Fig. 1:**
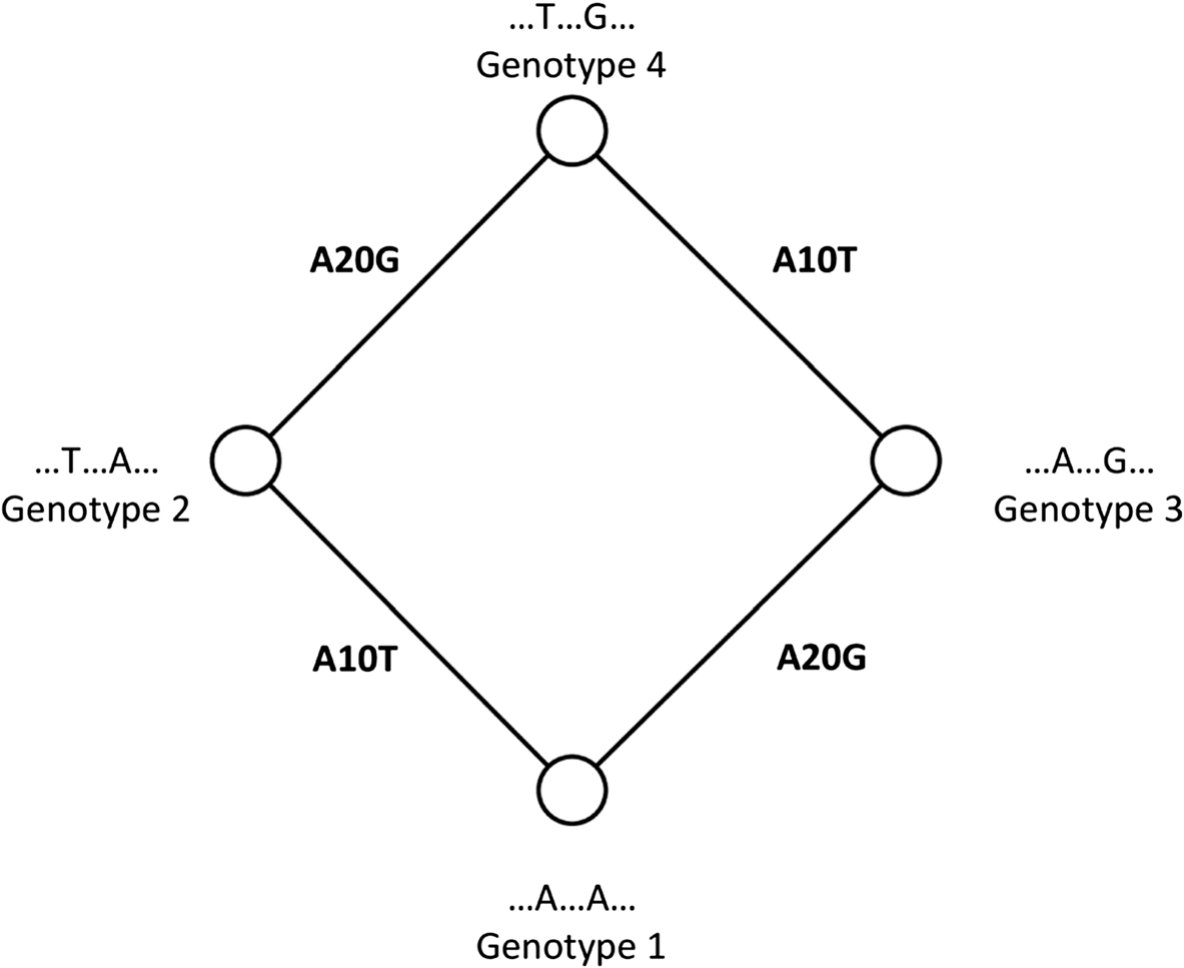
A hypothetical example of a four-node cycle in a haplotype network. The example indicates a hypothetical DNA sequence where two nucleotide changes occur at position 10 and 20. Circles (nodes) correspond to genotypes. An edge connects two nodes if they differ by a single mutation. Lettering next to each node indicates the nucleotides at which two genotypes differ. Edge labels show changes required to create a genotype from its neighbor, e.g., “A20G” indicates a change from A to G at position 20 of the hypothetical sequence. In the example, mutations at positions 10 and 20 create genotypes 2 and 3 from genotype 1 . Then, either genotype 2 mutates at position 20 from A to G, or genotype 3 mutates at position 10 from A to T, or both of these mutations take place, and create genotype 4

Figure 1 shows a short cycle in a hypothetical genotype network involving four DNA sequences. Edges reflect adjacent genotypes that differ in a single nucleotide. Assume, for example, that genotype 1 is ancestral to the other genotypes, and different substitutions (A10T and A20G) produce genotypes 2 and 3 from it. Genotype 3 then experiences an additional A10T substitution that creates genotype 4. This mutational path leads to a closed cycle, where three of the four edges reflect a substitution event. The fourth edge is a consequence of the first three events, because they render genotype 2 adjacent to genotype 4. Similar scenarios can be developed if a genotype different from genotype 1 is ancestral. Regardless of this ancestor, cycles require sequence changes that render the descendants of one (or more) genotypes more similar rather than less similar. In other words, cycles require some form of homoplasy, i.e., parallel or convergent evolution [28–32]. More generally, homoplasy is said to exist when two lineages display the same genetic or phenotypic characters, even though this similarity has not arisen through common ancestry [28, 32].

Homoplastic sequence evolution has been documented in a wide variety of molecules [33–39]. It can be caused by chance alone, which is expected to be rare in long evolving biopolymers with multiple kinds of monomers, because random mutations are more likely to cause such polymers to diverge than to converge. Mutational biases, strong selective constraints on sequence evolution [37], positive selection [33–37], or genetic recombination [40] can also cause homoplasy.

Here we construct haploid genotype networks for each of 12,235 genes in the human genome, based on single nucleotide variation data available for 1,092 individuals from four continents [10]. We analyze short cycles up to length eight in these networks, and find that the haploid genotype (haplotype) networks of 42 genes show a significant excess of cycles that cannot be explained by chance alone. After having excluded recombination as a prominent cause of these cycles, we focus on positive selection as a possible cause, and present evidence that in at least some of these genes positive selection may help explain the existence of cycles.

## Results

### Constructing and characterizing haplotype networks

To construct genotype networks for 1,092 human individuals, we used haploid genotypes (haplotypes) with single nucleotide variation data available from the 1,000 genomes project [10]. Thus, our genotype networks are haplotype networks, and from now on, we use the term haplotype network instead of genotype network. For each human gene, we constructed one haplotype network. Two principal definitions of such a network are germane for this paper. By the first definition, a haplotype network of a human gene is a graph whose nodes correspond to protein-coding DNA sequences of the gene in different individuals. Two nodes (sequences) are adjacent if they differ in a single base pair (i.e., by either a synonymous or non-synonymous change). By the second definition, two nodes are adjacent if their coding sequences differ by a single non-synonymous (amino acid replacement) change. The second kind of network can thus also be viewed as a network of proteins or amino acid sequences, in which neighboring proteins differ in a single amino acid.

**Fig. 2:**
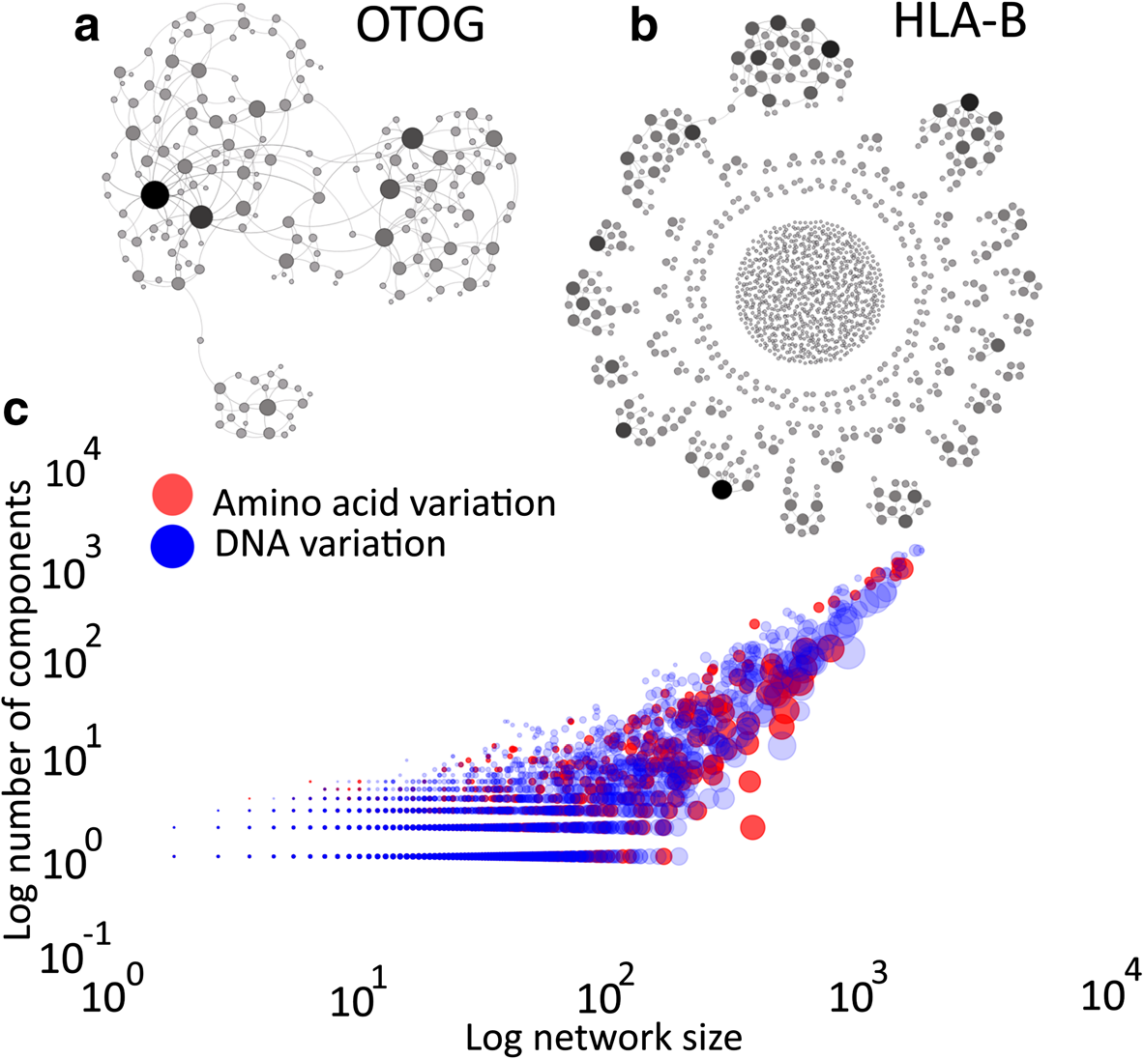
Haplotype networks vary greatly in structure among genes. **a** Haplotype network of the gene *OTOG* (Otogelin). Among all protein-based haplotype networks comprising more than 100 sequences, *OTOG* has the network with the largest giant component where all nodes fall into this component (181 nodes and a single component). **b** Haplotype network of the gene *HLA-B*, which is the most fragmented network, with 1,545 nodes in 1,111 components. Circles in **a**) and **b**) correspond to different genotypes, while edges connect genotypes that differ by a single point mutation. Circle color and size correspond to the degree (number of neighbors) of the node, where darker and larger nodes have a higher degree. **c** Number of components versus network size for DNA-based (*blue circles*) and protein-based haplotype networks (*red circles*). Circle size in **c**) corresponds to the relative size of the giant component within each haplotype network

We first created both DNA- and protein-based haplotype networks based on the above definition, collapsing those nodes with identical sequences into one (see Methods). Networks can be made of one or more components. Each component is a subgraph in which any two nodes are connected to each other by a path of edges. We found that the average size of the largest connected component – commonly referred to as the giant component – relative to total network size is significantly larger in protein-based networks (12,235 proteins, a fraction 0.975 of the total network) than in DNA networks (15,841 DNA sequences, 0.940 of the total network) (Mann–Whitney U test – *p*-value = 7.01e-156) (See also Fig. 2c). Because our statistical analyses focus on the giant component of each haplotype network and work best if this component comprises as many nodes as possible, we focus on protein-based haplotype networks for the rest of this contribution. The 1,000 genomes dataset we use contains information from 19,744 genes, but we constructed haplotype networks only for those 12,235 protein-coding sequences that showed at least one amino acid variant.

Figure 2a and b illustrate with two examples that haplotype networks for different genes can differ greatly in their topology. The left network (Fig. 2a), derived from the gene *OTOG*, which encodes Otogelin, comprises 181 nodes organized into a single component, whereas the right network, from gene *HLA-B* (Major histocompatibility complex, class I, B) is highly fragmented and has 1,545 nodes in 1,111 different components (See Additional file 1: Figure S1 for a different representation of the two networks.)

More generally, Fig. 2c shows the distributions of the number of components and the size of the largest component for all genes we considered. There are 11,155 networks with only a single component, but most of these networks are small, with an average of 5.52 sequences. The network with the most components is the highly fragmented *HLA-B* network with 1,111 components. *HLA-B* is known to be under strong balancing [41] and divergent selection [42], which causes great genotypic diversity. This diversity translates into high network fragmentation, i.e., a network with many components. Some haplotype networks have very large giant components with up to 552 sequences. However, in most (10,587) networks, the largest component is very small, comprising a maximum of ten sequences. The network with the largest giant component where all sequences fall into that component is that of *OTOG* (Fig. 2a).

### Cycles in haplotype networks

A cycle in a network is a series of edges starting from one node and ending with the same node, while passing other nodes along the cycle only once. In haplotype networks constructed from biallelic gene variants, the simplest elementary cycle, i.e. a cycle not decomposable into smaller cycles, is a square. The reason is that cycles with an odd number of edges, e.g. triangles or pentagons, are impossible when all SNPs are biallelic. Figure **1** shows a square that involves the mutation of a hypothetical DNA sequence at two different sites (positions 10 and 20). Next to each circle (sequence) the nucleotide residues at these positions are indicated, and along the edges, the specific nucleotide changes that occurred. If genotype 1 is the most recent common ancestor of its neighbors, then its two neighbors have undergone two different mutations: Specifically, genotype 2 has experienced a change from *A* to *T* at position 10 and genotype 3 has a change from *A* to *G* at position 20. To form the single genotype 4 from its ancestors, i.e. from either genotype 2 or 3, either genotype 2 has undergone a change from *A* to *G* at position 20, or genotype 3 has undergone a change from *A* to *T* at position 10, so that the descendants of the two ancestral sequences 2 and 3 become not only more similar but identical to one another. It is not necessary for both of sequences 2 and 3 to mutate to form genotype 4, but a mutation in either of them can lead to the genotype and form a cycle. Regardless of whether genotype 1 or any other genotype is the common ancestor of the others, a square like this requires convergent sequence change.

In long biopolymer sequences with multiple monomers that evolve through random mutation alone, cycles should be rare, because it is unlikely that mutations become reversed to create sequences more similar to one another. However cycles can be introduced by mutation biases that allow only certain residues to change, or by selection that causes only certain changes to survive, i.e., by evolutionary constraints. Another possibility is recombination, which might occur between genotypes 2 and 3, which can result in genotype 4. The same mechanisms can give rise to longer cycles (e.g., length 6 or 8, Additional file 2: Figure S2).

**Fig. 3:**
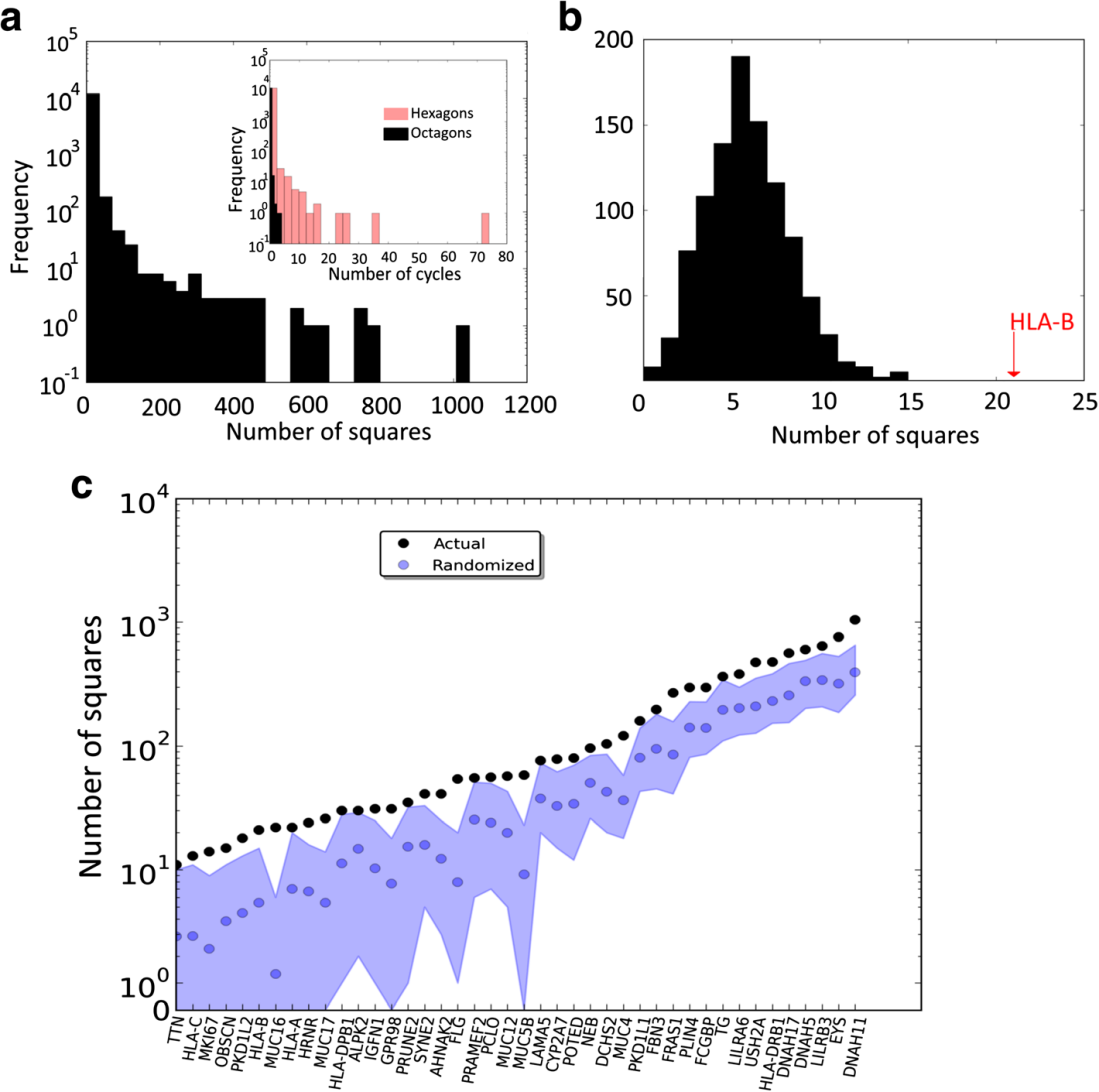
Distribution of the number of cycles in all networks and in networks with an excess of squares. **a** Distribution of the number of elementary squares, as well as elementary hexagons and octagons (inset) in all protein-based networks. **b** Distribution of the number of squares in 1,000 randomized networks derived from the giant component of the *HLA-B* network, whose number of squares (21) is indicated by a red arrow. **c** Number of squares (*black circles*) in the largest components of the haplotype networks of 42 genes with significantly more squares than expected by chance alone, together with the mean number of squares (*blue circles*) found in 1,000 randomly generated networks for each gene. Shaded areas depict the maximum and minimum number of squares in the randomized networks. Note the logarithmic scale on the vertical axis.

Figure 3a shows the distribution of the number of squares in all networks. 7,373 of 12,235 networks had no squares. The network with the most squares is that of the gene *DNAH11* and contains 1,043 squares. The inset of Fig. 3a shows the distribution of hexagons and octagons. The networks with the largest number of hexagons (74) and octagons (4) are those of genes *MAP2K3* and *HLA-B*, respectively. Note the small numbers of hexagons and octagons compared to squares. Even though we enumerated elementary cycles up to length eight – beyond that, our analyses become computationally too costly – we focus most of the following analysis on squares, because they are by far the most abundant cycles.

### Unconstrained or constrained mutation cannot explain the large number of cycles in many networks

Because some amount of homoplasy can occur by chance alone, we wished to determine whether all squares we observed could have occurred by chance homoplasy. To this end, we created randomized haplotype networks in which the same amount of evolutionary change occurred as in the actual networks.

In our first randomization procedure, we created a (simulated) DNA sequence of the same length as the coding sequence of a gene, and created a haplotype network from it by simulating a pattern of mutations designed to yield a network with the same number of edges (number of nonsynonymous changes) and the same distribution of degrees (number of neighbors) as the actual network (see Methods). Specifically, we compared the number of squares in each haplotype network to 1,000 such randomly generated networks, and found 4,267 genes whose actual number of cycles was greater than all of the 1,000 randomly generated networks. Thus, based on this criterion there are 4,267 genes whose total number of squares cannot be explained by chance homoplasy alone (*p*-value ≈ 0.001 – FDR ([43]) at 0.05) (a full list of these genes can be found in Additional file 3). 

One can argue that this procedure does not take into consideration the actual patterns of variation observed in the data, namely that only a small subsets of sites in any one gene have been subject to mutation, and that all of the sites are biallelic, that is, only two variant nucleotides occur in them. Both patterns arise from the fact that the human population sample is not highly diverged, and that natural selection constrained the evolution of these sequences, i.e., it eliminated some mutations that occurred in them. We thus modified our randomization procedure to reflect these facts (see Methods). With these more conservative criteria, we still found 42 genes (0.34 % of all genes analyzed) whose haplotype networks have significantly more squares in their networks than expected by chance alone (Table 1). That is, their number of squares cannot be explained by mutational patterns and purifying selection alone. Figure 3b shows, as an example, the number of squares (21, orange arrow) in the haplotype network of *HLA-B*, which is 6.52 standard deviations greater than the mean number of cycles (5.36) in 1,000 randomized networks (black histogram). Figure 3c shows the number of squares in all 42 networks (black circles), together with the mean (blue circles), minimum, and maximum (blue shaded regions) number of squares for 1,000 randomized networks created for each of the 42 haplotype networks.

**Table 1:** Genes with an excess of squares in their giant component

Gene name	Previous evidence of positive selection	Number of squares in the giant component
*TTN*	None	11
*MKI67*	None	14
*OBSCN*	None	15
*PKD1L2*	None	18
*MUC16*	None	22
*MUC17*	None	26
*IGFN1*	[54]	31
*GPR98*	[54]	31
*PRUNE2*	None	35
*SYNE2*	None	41
*AHNAK2*	None	41
*HLA-DPB1*	[62, 78–81]	48
*ALPK2*	None	50
*HLA-C*	[62, 78–81]	50
*FLG*	None	54
*PRAMEF2*	[54]	55
*HRNR*	None	55
*MUC5B*	None	58
*PCLO*	[54]	67
*HLA-A*	[62, 78–81]	67
*MUC12*	None	71
*LAMA5*	[54]	76
*CYP2A7*	[82]	76
*HLA-B*	[62, 78–81]	76
*POTED*	None	80
*NEB*	None	96
*MUC4*	None	121
*PKD1L1*	None	160
*FBN3*	[54]	197
*DCHS2*	None	205
*FRAS1*	[54]	269
*PLIN4*	None	298
*EYS*	None	316
*FCGBP*	[54]	350
*TG*	None	365
*USH2A*	[54]	475
*LILRB3*	None	475
*LILRA6*	None	482
*DNAH17*	[54]	494
*HLA-DRB1*	[55, 62, 78–81]	507
*DNAH5*	[54]	602
*DNAH11*	None	1043

The number of squares in these genes cannot be explained by random homoplasy or mutational constraints. The middle column cites studies that provide evidence for positive selection, wherever such evidence is available. After FDR correction, the *p*-value of the statistical test comparing the actual number of cycles against that in 1,000 randomized networks (with random mutations and mutational constraints) is 0.087 for all genes

**Fig. 4:**
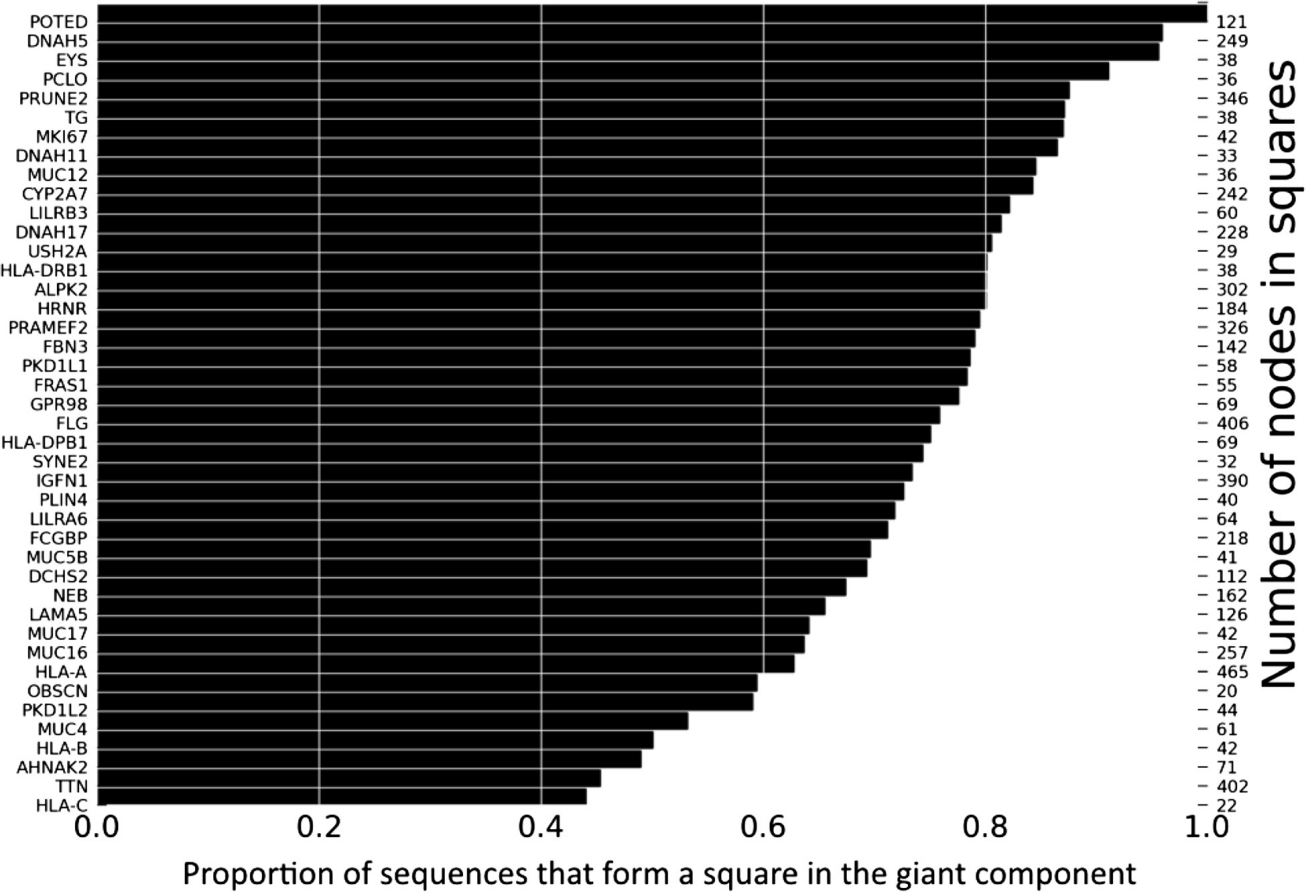
Proportion of sequences that are part of a cycle. Proportion (*horizontal axis*) and actual number of sequences (r*ight vertical axis*) that are part of a square in the giant connected components of haplotype networks for those 42 genes (*left horizontal axis*) with a significant excess of squares

Additional file 4: Figure S3 shows the distribution of elementary cycles with length four, six and eight among the 42 genes with an excess of squares, and Fig. 4 shows the proportion of the sequences that form part of a square in the largest connected component of each gene network. For some genes, such as *POTED* (POTE ankyrin domain family, member D) all sequences form part of a square, and even for genes where the proportion of sequences in a square is low, such as *HLA-C* (major histocompatibility complex, class I, C) and *TTN* (titin), it exceeds 40 % (Fig. 4).

We note that the 42 networks with an excess of squares are otherwise very heterogeneous in their properties. They range from the network of *MKI67* (marker of proliferation Ki-67) where only 23 nodes lie in the largest connected component, to the network of *DNAH11* (dynein, axonemal, heavy chain 11), where 538 nodes do (see Additional file 5: Figure S4 for the distribution of component sizes). Some of the networks have very few components, such as that of *POTED* with a single component, whereas others have many components, such as the highly fragmented *HLA-B* network with 1,111 components (see Additional file 6: Figure S5 for the distribution of component numbers). Even properties within the largest connected components are heterogeneous. For example, in some networks, such as that of *PKD1L1*, the distribution of the numbers of neighbors of each sequence is highly right-skewed and dominated by sequences with few neighbors, while in others it is more symmetric (*PRAMEF2*, Additional file 7). Assortativity coefficients, which quantify the tendency of each node to connect to other nodes with a similar number of neighbors, also vary broadly. Some networks are assortative (sequences with many neighbors are adjacent to other sequences with many neighbors), whereas others are disassortative (Additional file 8: Figure S7).

Gene Ontology (GO) enrichment analysis on biological processes shows several immune system-related processes that are enriched in the 42 genes, namely “antigen processing and presentation of endogenous peptide antigens” and “interferon-gamma-mediated signaling pathway” (see Additional file 3 for full results of the analysis and parameters). GO enrichment analysis of molecular functions reveals the two enriched functions “calcium ion binding” and “peptide antigen binding”. “Peptide antigen binding” is again associated with the immune system.

Given the strong representation of HLA genes among genes with an excess of cycles, we also asked how the GO enrichment analysis would change if we excluded the HLA genes. We found a single enriched biological process, namely “O-glycan processing”, and two enriched molecular functions, namely “calcium ion binding” and “extracellular matrix constituent, lubricant activity”.

We also asked whether genes with an excess of squares preferentially occurred in specific KEGG [44] or Reactome [45] pathways. Six genes were preferentially associated with KEGG pathways. They include TG (thyroglobulin) and the genes in the HLA family. The enriched pathways comprise “Epstein-Barr virus infection”, “Autoimmune thyroid disease”, “HTLV-I infection”, “Viral myocarditis”, “Allograft rejection”, “Phagosome”, “Antigen processing and presentation”, “Graft-versus-host disease”, “Cell adhesion molecules (CAMs)”, “Herpes simplex infection”, and “Type I diabetes mellitus”.

For Reactome pathways, we found twelve genes enriched in six pathways. The genes include those encoding Mucins, the HLA family and LILR family genes (MUC4, MUC5B, MUC12, MUC16, MUC17, HLA-A, HLA-B, HLA-C, HLA-DRB1, HLADPB1, LILRA6, and LILRB3). The enriched pathways are “Termination of O-glycan biosynthesis”, “Interferon gamma signaling”, “Endosomal/Vacuolar pathway”, “Immunoregulatory interactions between a Lymphoid and a non-Lymphoid cell”, “Antigen Presentation: Folding, assembly and peptide loading of class I MHC” and “Defective GALNT12 causes colorectal cancer 1 (CRCS1)”. We note that both enriched KEGG and Reactome pathways include several immunity-related pathways.

### Recombination cannot account for an excess of squares in most networks

To exclude the possibility that genetic recombination may account for the excess of squares in some networks, we performed two complementary analyses. First, we simulated for each gene the effect of recombination on haplotype network structure by creating haplotype networks based on a set of sequences that were subject to approximately as many recombination events as occurred in the human population since their common ancestry, as well as to as many mutations as there are edges in the network (see Methods). We repeated this process 1,000 times for each gene, creating 1,000 simulated haplotype networks, and counted the number of squares in them. For each of the 42 genes, the empirical network showed more squares than each of the 1,000 simulated networks (Additional file 9: Figure S8).

In the second analysis, we asked whether gene conversion, a process of unidirectional recombination in which only one of the recombining sequences changes, may have caused the excess of squares [46, 47]. To this end, we used the program GENECONV (version 1.81a) [48] to detect gene conversion among the sequences in the giant components of the 42 haplotype networks. We used sequences comprising both synonymous and non-synonymous changes to give the program more power in finding gene conversion events. Only one gene showed any sign of gene conversion, and it did so for only two of 114 sequences in *CYP2A7* (cytochrome P450, family 2, subfamily A, polypeptide 7). In sum, based on these analyses, it seems unlikely that recombination can explain the excess of squares we observe in the haplotype networks of 42 genes.

### Positive selection as a potential cause of squares

Positive selection can be a driver for homoplastic or convergent evolution, where two separate lineages evolve the same trait independently [49]. Because such adaptive homoplasy can occur not only at the phenotypic level [50, 51], but also at the sequence level [34, 35, 52], we wished to find out whether positive selection can help explain the excess of squares we observed in the haplotype networks of 42 genes.

Previous studies had indeed indicated positive selection for at least 17 of the 42 genes (Hughes and Nei 1988, 1989; Ohta 1991; Hughes and Yeager 1998; Birtle et al. 2005; Crespi and Summers 2006; Proux et al. 2009; Kawashima et al. 2012) (Table 1). In addition, we used results from a branch-site likelihood test [53] which indicates positive selection based on a ratio dN / dS > 1 observed along one or more branches of a phylogenetic tree. This test has been applied to vertebrate genes in the Selectome database [54], which indicates that 12 of our 42 genes with abundant squares show patterns of positive selection, either in primates or in the bony vertebrates (Euteleostomi, Table 1 and Additional file 10: Table S2). This number – 12 of 42 – is unlikely to be observed by chance alone (*p* = 0.0004; hypergeometric test, based on 2,125 unique genes in the human genome under positive selection according to Selectome (data provided by the authors of Selectome)). In addition, we used the XP-CLR (cross-population composite likelihood ratio) test for neutrality [55] (see Methods). The test compares different populations to identify rapid changes in a locus’ allele frequency that cannot be explained by random drift alone. In applying this test, we used a test statistics [56] pre-computed over 2 kb sliding windows that covered the human genome, and asked for each of our 42 genes whose haplotype network showed an excess of squares, whether two or more of the windows where the test-statistic indicated the action of positive section (*p* = 0.01) overlapped with the gene (see Methods). By this criterion, six of our 42 genes showed evidence of positive selection in at least one population (Additional file 11: Table S1 and Additional file 12: Table S3). Overall, 21 of our 42 genes with an excess of squares showed signs of positive selection by at least one of these criteria or by previous work.

We also analyzed patterns of synonymous and non-synonymous changes in more detail. A commonly used indicator of positive selection for two protein-coding DNA sequences is d_N_ / d_S_, i.e. the ratio of nonsynonymous changes d_N_ per nonsynonymous site to synonymous changes d_S_ per synonymous site. Values of d_N_ / d_S_ > 1 can indicate positive selection [57, 58]. Unfortunately, d_N_ / d_S_ can be computed only for sequences more distantly related than those in our haplotype networks. The reason is that in these networks, adjacent sequence pairs differ only in a single nonsynonymous mutation, and many adjacent pairs do not even show a single synonymous change. More specifically, in the giant component of our networks, up to 80 % of sequence pairs do not show a single synonymous mutation (Additional file 13: Figure S9), and this incidence of synonymous mutations is similarly low in the entire network. Moreover, it has been suggested that for very closely related sequences, d_N_ / d_S_ is not a sensitive indicator of positive selection [59]. For these reasons, we compared the incidence of nonsynonymous and synonymous changes among groups of edges (see Methods), reasoning that groups of edges with very few synonymous changes might provide hints that some or all members of the group may have been subject to positive selection. Most edges show no synonymous changes at all in some networks, which hints that positive selection may have played a role in creating their pattern of diversity (Additional file 13: Figure S9).

**Fig. 5:**
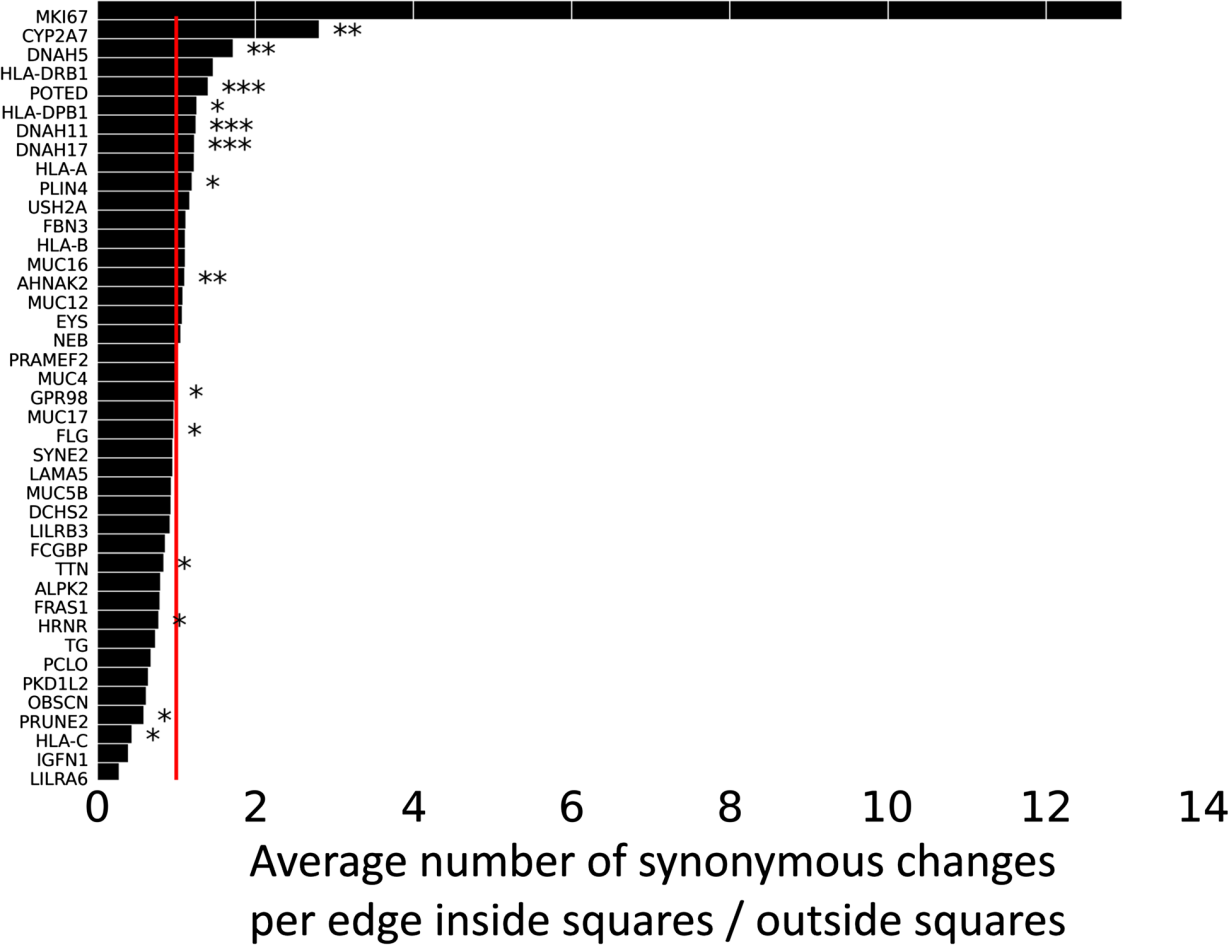
Ratio of the average number of synonymous changes per edge for edges inside squares relative to edges outside squares. The red line corresponds to a value of this ratio that is equal to one, i.e, edges inside and outside squares have the same average number of synonymous changes. Bars that end to the left (*right*) of this line indicate genes in which the average number of synonymous changes per edge is lower (*higher*) inside squares than outside squares. *, **, and *** indicate that the difference between the average number of synonymous changes inside versus outside squares is significant at *p*-values of 0.05, 0.01, and 0.001, respectively (Mann-Whitney U test). The *p*-values are corrected following [43]

We specifically compared edges with no synonymous change inside squares and outside squares. While the fractions of edges without synonymous changes inside squares was not significantly different from those outside squares (Fisher’s exact test on 2 × 2 contingency tables, Additional file 14: Figure S10), the average number of synonymous changes on edges inside squares was significantly smaller than that outside squares for 14 % (6) of the genes (Mann-Whitney U test, *p*-value = 0.05, FDR corrected). Figure 5 shows the average number of synonymous changes per edge for edges inside squares divided by that for edges outside squares. For genes where this ratio is below 1 (red vertical line) the average number of synonymous changes are smaller inside squares than outside squares. Overall, the distribution of synonymous changes among edges inside squares and outside squares does not suggest that all incidences of excessive squares are due to positive selection, but it suggests that positive selection may have contributed to this excess for at least some genes.

Using a test based on the hypergeometric distribution [60], we found no significant overlap between the genes that showed evidence of positive selection in the XP-CLR test and those genes among our 42 focal genes that (i) have significantly fewer synonymous mutations inside the squares than outside the squares of their haplotype network (2 common genes) or (ii) had been identified in several previous studies as being subject to positive selection (3 common genes).

### Balancing selection is not a likely cause of an excess of squares

**Fig. 6:**
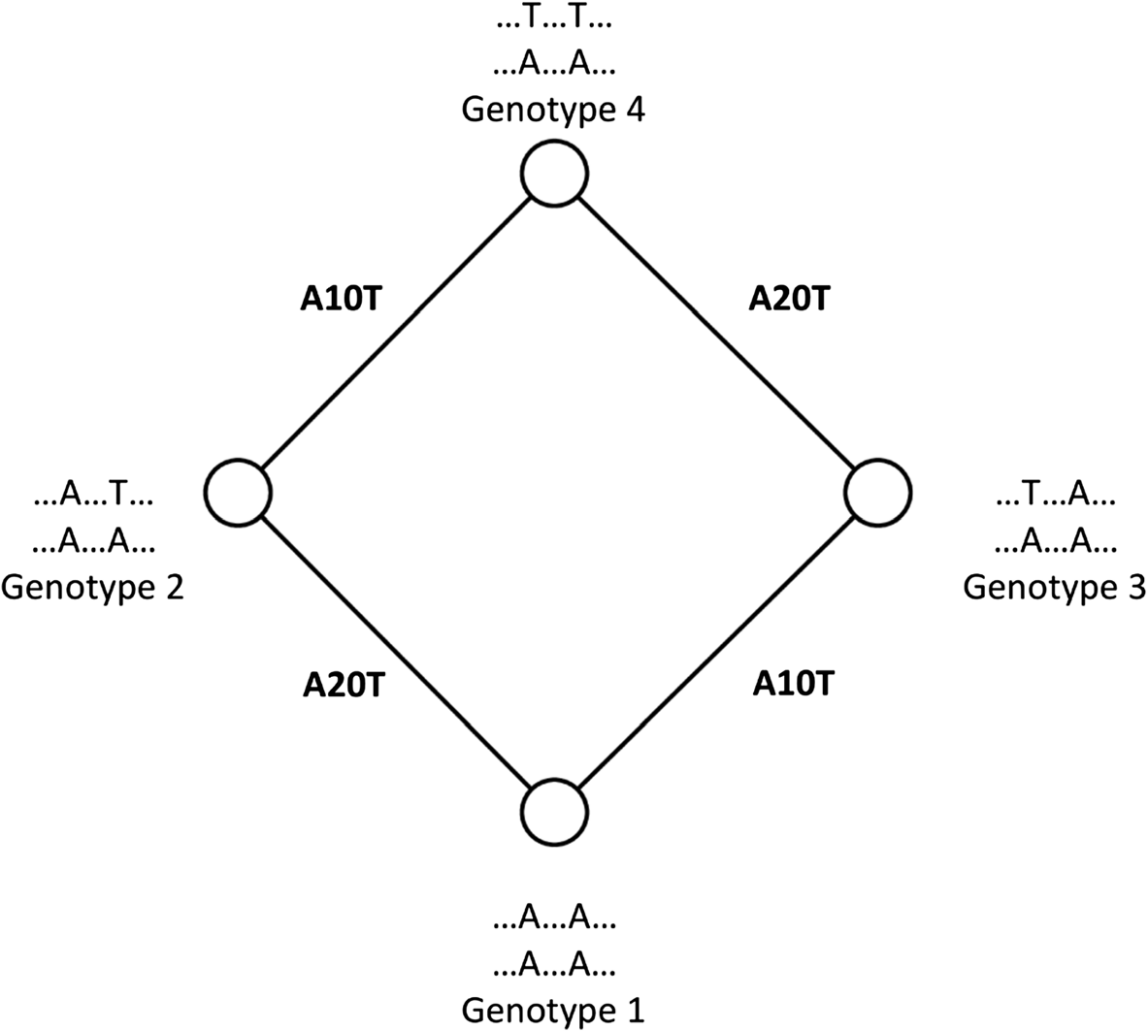
Balancing selection can produce cycles. The example indicates a hypothetical diploid genotype where two nucleotide changes occur at position 10 and 20. Circles (nodes) correspond to genotypes. An edge connects two nodes if they differ by a single mutation. Lettering next to each node indicates the nucleotides at which two genotypes differ. Edge labels show changes required to create a genotype from its neighbor, e.g., “A20G” indicates a change from A to G at position 20 of the first haplotype of the hypothetical genotype. See text for details

In a final analysis, we also asked for evidence of balancing selection, which manifests itself as an elevated amount of heterozygosity and can in principle produce squares. Consider, for example, the square in Fig. 6, in which nodes represent hypothetical diploid genotypes. Next to each circle (genotype) the nucleotide residues at positions 10 and 20 are indicated, and along the edges, the specific nucleotide changes that occurred for the first of two haplotypes. If genotype 1 is the most recent common ancestor of genotypes 2 and 3, then a substitution at site 20 in the first haplotype of genotype 1 creates genotype 2, and a substitution at site 10 of the first haplotype creates genotype 3. If balancing selection is acting on both sites (10 and 20), individuals 2 and 3 will be favored over individual 1, because they are heterozygous at one of the two sites under balancing selection. A further substitution to genotype 4, would create a double-heterozygous genotype – and a square – that is even more favored by balancing selection.

We computed for each gene the fraction of heterozygous individuals averaged over all sites that experienced nonsynonymous changes in at least one individual of the sample population (see Methods). Among our 42 genes with an excess of squares, we found no significant (Pearson’s r, *p*-value = 0.512) correlation between the number of squares and heterozygosity. For all 19,744 genes, we found a very small (Pearson’s *r* = 0.066) yet significant correlation (*p* = 3.42 × 10^-13^) between heterozygosity and the number of squares in a gene’s haplotype network (Additional file 15: Figure S11). In sum, balancing selection is not a likely explanation for the prevalence of squares in some genes.

### Multiple genes whose haplotype networks show an excess of squares are implicated in immune functions

Especially prominent among the 42 genes whose haplotype networks show an excess of squares are genes with immune functions. Such genes are also known to be subject to frequent positive selection [61]. For example, five of the 42 genes belong to the human leukocyte antigen (HLA) family. These are the genes *HLA-A*, *HLA-B*, *HLA-C*, *HLA-DPB1*, and *HLA-DRB1*. HLA genes show the highest level of polymorphisms in the human genome [2, 62], and display multiple signatures of positive selection, including a high d_N_ / d_S_ in antigen-recognition sites, trans-species polymorphisms, high levels of heterozygosity, as well as long range haplotypes, a key signature of recent positive selection [62].

Five more among the 42 genes with an excess of squares encode mucins, which are important for the immune response, because they help form mucus that can prevent pathogen entry, and cooperate with antibodies to fight pathogens [63–65]. These are *MUC4*, *MUC5B*, *MUC12*, *MUC16* and *MUC17*.

Two more among the 42 genes, *LILRB3* and *LILRA6*, encode leukocyte immunoglobulin-like receptors, which cooperate with MHC proteins. LILRB1, another member of this family, has co-evolved with HLA, which is under positive selection in sub-Saharan population [66]. Another immune-relevant gene among the 42 genes is *FCGBP*, which may play an important role in immune protection and inflammation in the intestines of primates [67].

## Discussion

We show that the haplotype networks of 42 genes display a significant excess of squares that cannot be explained by chance homoplasy, genetic recombination, or balancing selection alone. This leaves constrained evolution as a prominent candidate cause, which limits the diversity of alleles that are generated or preserved in a sequence. While such constrained evolution can have multiple causes [68], strong purifying and positive selection are most relevant for the kind of data we analyze.

Strong purifying selection may play a role in the occurrence of squares, because we observed significantly fewer squares for many genes in our randomization tests when we allowed the whole protein coding sequence to change, and when we permitted substitutions to any nucleotide. In addition, some of the genes with an excess of squares may have experienced positive selection. First, up to 80 % of edges in the giant component of some of these genes do not have any synonymous mutations at all (Additional file 13: Figure S9). Second, six of the genes with an excess of squares (14 %) have significantly more synonymous changes outside their squares than inside them (Fig. 5). Third, six genes contained at least two adjacent windows with a significantly high value of the XP-CLR test statistic that can indicate positive selection (Additional file 11: Table S1). Fourth, previous studies have suggested that 17 of the 42 genes with an excess of squares have been subject to positive selection (Table 1). Finally, multiple genes among those with an excess of squares are involved in immune functions, which are frequently subject to positive selection [61].

More generally, it is relevant that there is a mounting number of known genes where convergence at the sequence level has been caused by positive selection. For example, sequence convergence occurred in the peptide-binding regions of human and mouse class Ib genes in the major histocompatibility complex (MHC), the same gene family in which five members show an excess of squares in our study [39]. The motor protein Prestin which is involved in the mammalian auditory system has experienced adaptive sequence convergence between echolocating bats and echolocating dolphins [33]. Two other genes involved in the mammalian auditory system, Tmc1 and Pjvk, also have experienced convergence due to positive selection [37]. In addition, whole genome sequencing of four bat species showed extensive genome-wide convergence among these taxa [34]. Moreover, extensive convergent evolution occurred between snake and agamid lizard mitochondrial genomes, much of which may be adaptive [35].

Our analysis is based on some 1,000 human genomes, which raises the question how its results might be affected as the size of the available data set increases. Most importantly, a larger data set would lead to larger and more connected networks. Our analysis is focused on the largest connected component of each network, and increasing the size of the largest connected component could lead to more cycles just by chance alone. Indeed, larger connected components of a haplotype network in our data set also contain more cycles (Additional file 16: Figure S12). This pattern also extends to those networks with a significant excess of cycles. Specifically, giant component sizes are significantly larger for networks that have a significant excess of cycles than for the remainder of the haplotype networks (Additional file 17: Figure S13). Conversely, a higher fraction of genes with an excess of cycles have large giant components (>100 nodes). These observations suggest that increasing the size of our data set might not just increase the overall number of cycles, but also the number of haplotype networks with an excess of cycles. In other words, it would increase the sensitivity of our analysis.

A recent study [69] has shown that HLA genes show reference allele bias in the 1,000 genomes data. Removing these alleles from the dataset could in principle lead to smaller giant components in the HLA networks and hence to fewer cycles. However, this is unlikely to materially affect our observations, because the largest components, with one exception, comprise a small fraction of the HLA networks (0.05, 0.26, 0.09, 0.60 and 0.04 for HLA-B, HLA-DPB1, HLA-A, HLA-DRB1 and HLA-C, respectively). Thus, most removed alleles would fall into other components, and their removal would thus not affect our giant-component-based analysis.

In sum, while we have not been able to explain the abundance of squares conclusively, we suggest that a mix of constrained evolution through purifying selection and positive selection may be responsible. As data from more and more individuals from the global human population become available, it will be possible to disentangle these causes. Such data may also help explain the great differences in haplotype network structure among the human genes we characterized here.

## Conclusions

We explored a novel way of representing human genetic variation data through a network-based approach whose strengths are complementary to phylogenetic trees. Despite the fact that the genes in the genomes we analyze have a shared phylogenetic history, they show very diverse properties in their haplotype networks. Specifically, these networks show different numbers of genotypes (Fig. 2c), different extents of fragmentation (Additional file 6: Figure S5), different degree distributions (Additional file 7: Figure S6), and different assortativity (Additional file 8: Figure S7). Our analysis focuses on the feature of these networks that cannot be easily represented in phylogenetic trees, i.e., cycles. Phylogenetic trees are acyclic, and thus not ideally suited to represent evolutionary histories more complex than direct descent, such as allopolyploidization, convergent evolution, sexual reproduction, recombination and horizontal gene transfer. Such events can transform a tree-like evolutionary history into a reticulate network. Haplotype networks can represent such reticulation, and can thus complement phylogenetic trees in their ability to represent and describe evolutionary processes.

## Methods

### Construction of haplotype networks

We focused our analysis on haplotype networks built from amino acid changing (non-synonymous) mutations of all genes in the human genome, and supplemented this analysis with data on synonymous mutations. The data we use consists of SNPs called from sequencing of 1,092 individuals by the 1,000 genomes project phase I [10]. First we downloaded variant call format (VCF) files [70] containing all genotypic variants for all 1,092 individuals, as well as the functional annotation of the variants (build 23.11.2010) provided by the 1,000 genomes project. At this stage we had 22 VCF files, one for each of the 22 autosomal chromosomes.

Next, using the software VCFtools [70], we filtered the VCF files by removing all sites with a “FILTER” tag other than “PASS”, as well as indels, non-phased variants, and all variants with a minor allele frequency smaller than 0.01. Analyzing VCF files after filtering, we found no SNP with more than two alleles, which is why all our analyses are based on biallelic SNPs. Subsequently, we used the previously obtained functional annotation information to create three VCF files for each gene, which contained nonsynonymous, synonymous, and both synonymous and non-synonymous SNPs in the gene’s protein coding region.

The networks we analyze are built on the basis of haplotypes, i.e., we considered for each individual its two haploid genotypes separately. Each network is a graph whose nodes are haplotypes, and two haplotypes are connected by edges if they differ in a single SNP. Overall, we analyzed 2,184 haplotypes, and established a separate haplotype network for each of 17,744 human genes. We constructed and analyzed all networks with the help of the iGraph package for Python (version 0.6.5) [71], and visualized them using Gephi (version 0.8.2-beta) [72].

For our analysis of protein-based haplotype networks, we merged two haploid genotypes into a single node of the network if they had identical haplotypes based on their non-synonymous SNPs. Some of our analyses required us to compute the number of synonymous changes between adjacent nodes of these networks, and because a node does not necessarily correspond to a unique haplotype, this number is also not unique – different haplotypes encode the same protein but they may differ at synonymous sites. Wherever this was the case, we used in our analysis the average number of synonymous changes along an edge, computed by enumerating synonymous changes between all possible pairs of haplotypes for the incident nodes.

### Analysis of cycles and other network properties

Cycles in a haplotype network are paths that start and end at the same node, while visiting every other node in the path exactly once. We note that in a haplotype network of biallelic SNPs, no cycles of uneven length are possible. We first focused on cycles of length four, i.e., squares, and calculated their number through exhaustive enumeration. Specifically, we started from any one node and walked from there to all its neighbors, the neighbor’s neighbors, and so on, avoiding previously visited nodes, until we had visited five nodes. Any sequence of five nodes is a square if the first and last nodes in the sequence are identical. Repeating the same procedure from all nodes in the network allowed us to enumerate all squares (not double-counting squares that we had found more than once). We applied the same approach to find longer cycles of length six and eight. We call such a longer cycle elementary, if it is not decomposable into shorter cycles, and we verified this property for each longer cycle.

### Randomized haplotype networks

To ask whether the number of cycles in an empirically observed haplotype network is greater than expected by chance alone, we created randomized haplotype networks for each gene. More specifically, this analysis focused on the largest component of each gene’s haplotype network, which comprises on average 97.5 % of a network’s nodes.

A randomized network may have fewer or more cycles than the actual network. Consider the hypothetical square *uvyw* in a haplotype network, where *v* and *w* are located at two diagonally opposed corners of the square. In creating a random network, we might start from a node (sequence) *u*, mutate the sequence twice at random to create nodes *w* and *v*, and then mutate *w* and *v* once more (into *w’* and *v’*), so that we have created a random network of four edges. If *w’* and *v’* are not identical to each other and to the sequence *y* in the square this random network is not cyclic, whereas the actual four-node network is. (The opposite is also possible, where the randomization process creates a cycle where the actual network does not contain one.)

We performed two types of randomization analyses, one only with mutation and the other with mutation and recombination. Before we explain these analyses, we highlight a methodological detail. As we mentioned in the introduction of the paper, three substitutions are necessary to observe a square (and four are possible). In our randomization analyses described below, we always use four mutations, which is a statistically conservative choice. It allows edges in randomized networks that have no corresponding edge in the data-based networks, and some of these edges can lead to the creation of additional cycles. Thus, the number of cycles expected by chance alone (i.e., in randomized networks) will be somewhat higher with our procedure than in a population evolving subject to the assumptions we make below. This renders any assertion that a haplotype network contains more cycles than expected by chance statistically conservative.

#### Randomization with mutation

In a first randomization analysis, we aimed to create, for each gene, networks with the same number of nucleotide changes as the gene’s actual network. To construct such a random network, we began with a single random sequence that we then mutated iteratively. Specifically, we chose a random node *u* from the actual network and assigned a random sequence to it. Then we mutated the sequence as many times as *u* had neighbors in the actual network, and assigned each mutated sequence to one of the neighbors. Next, we cycled over each of these neighbors, and for each such neighbor *v* we mutated its assigned sequence as many times as the number of neighbors *v* had in the actual network. We repeated this simulated mutation process until all nodes in the original network had been visited, and for as many mutations as there were edges in the original network, thus creating a random network based on the same number of edges as the original network. Overall, for each gene we created 1,000 such random networks, and counted the squares in all of them.

In this process, we used two different kinds of starting sequences. The first was a random DNA sequence with the same length as the full length protein coding DNA sequence, where each of the four nucleotides was equally likely to occur at every site. Because most human genes have multiple transcripts and the transcripts may overlap with each other, we considered the total length of a gene’s protein coding DNA as the stretch of DNA that was covered by at least one transcript. We allowed every site to mutate into one of the three other nucleotides, as long as the mutation was nonsynonymous. To create nonsynonymous mutations, we chose a transcript for the gene at random, and mutated a random nucleotide site within that transcript. We mutated this nucleotide to some other randomly chosen nucleotide, and determined whether the change was nonsynonymous. If so, we kept the mutation, otherwise we repeated this procedure until we had found a nonsynonyomus change.

The second kind of starting sequence takes into account the observed pattern of variation in the sequences under consideration. This sequence comprised only as many nucleotide monomers as there were sites with nonsynonymous changes in a gene’s protein coding amino acid sequence. Moreover, since our data comprises only biallelic SNPs, we allowed each site in this sequence to convert only between two types of residues. We note that relaxing either assumption would lead to even fewer squares in a randomized network than we found. Thus, a randomization test based on this starting sequence is highly conservative.

Since more than 1,000 randomization tests for each network were not computationally feasible, the *p*-values of our tests could not be smaller than 0.001. To correct for multiple testing, we first assigned a *p*-value of 0.001 to those networks that had more squares than each of their corresponding 1,000 randomized networks. Then we adjusted *p*-values of all the networks that had at least one square (4,862 networks) using the procedure of Benjamini and Hochberg [43]. When building networks from full-length protein coding sequences, and from shorter sequences that reflect only the number of polymorphic sites, the adjusted *p*-values of genes whose randomized networks never had as many or more squares than the actual network were *p* = 0.001 and *p* = 0.087, respectively.

#### Randomization with recombination

To assess whether recombination can help explain the number of squares in human haplotype networks, we constructed, for each gene, 1,000 randomly generated networks that incorporate recombination during their construction, and determined the distribution of squares in these networks. To build a random network with recombination, we started with a collection or “population” of diploid sequences, whose size was half of the number of sequences in the giant component of the focal gene’s haplotype network. (We chose this size because we conceive of these sequence pairs as diploid “individuals” from which we would later construct a random haplotype network.) All individuals started with the same homozygous randomly generated sequence pair, which was as long as the number of nonsynonymous polymorphic sites in the gene. For each such sequence pair, we determined a number of mutation and recombination events that they were to undergo, as described further below. We then mutated each individual and recombined the two copies of its genome as many times as specified by these numbers. Subsequently, we randomly paired individuals and created each of two “offspring” from each pair by randomly sampling (with replacement) a haplotype from each parent in the pair to an offspring. We used these offspring to construct the random haplotype network, connecting two haplotypes if they differed by a single nonsynonymous mutation.

In this procedure, we wanted to generate a total number of mutations (for all sequences in the population) that was equal to the number of edges (nonsynonymous changes) in the giant component of the focal haplotype network. To this end, we first determined the average number of mutations per individual *M* as the total number of desired mutations divided by the number of haplotypes in the population. If *M* was an integer, we mutated each individual exactly *M* times. If *M* was a decimal number and *M* <1, then we introduced a single mutation into the individual with probability *M,* and no mutation with probability 1-*M*. If *M* was a decimal number and *M* >1, then *M* lay in the interval (*k*,*k* + 1), where *k* is some integer. In this case, we introduced *k* + 1 mutations into the individual with probability *M-k,* and k mutations with probability 1-(*M-k)*. We introduced each mutation into each haplotype by choosing a random site from the sequence and changing its nucleotide. To keep the variational constraints imposed by biallelic variation at each site, we only allowed each nucleotide to mutate to one other nucleotide.

If two sequences were to be recombined in the simulation, then recombination took place after mutation, and occurred between haplotypes of each sequence pair. To recombine a sequence *v* with a sequence *w*, we chose a random position in the sequence, and then replaced all the sites after that position in sequence *v* with residues in sequence *w,* and also replaced all sites after that position in sequence *w* with residues in sequence *v*. If two sequences were to be recombined more than once (see below), we repeated this process.

We next describe how we determined the number of recombination events for each haplotype network, where we aimed at introducing as many recombination events as are likely to have taken place in a gene, based on available polymorphism data. We calculated the fraction *r* of sequence pairs to be recombined once for each gene and used it for all random networks to be created for that gene. To obtain *r*, we first multiplied the average per-generation recombination rate in the human genome (0.952 cM/Mb per generation, calculated based on data from [73]) with the number of generations since the sequences in our data set may have shared a common ancestor. To estimate this number of generations, we used the number of synonymous mutations observed in each gene in our data set. Specifically, we used the following relationship

1 $$ generations\kern0.37em to\kern0.37em  common\kern0.37em  ancestry = \frac{S}{L \times \upmu \times Ne} $$

where *S* in the numerator designates the observed number of synonymous sites for that gene (determined using the filtered VCF files from the 1,000 genomes data). In the denominator, *L* is the length of the gene, including introns, as retrieved from Biomart (version 0.7, [74]), *μ* is the average human mutation rate per nucleotide (1.1 × 10^-8^) [75], and *N*_*e*_ is the effective population size, for which we used a value of *N*_*e*_ = 10,000 [76].

After having computed the estimated number of recombination events for each gene, we divided this number by the sample size of our data (1,092) to obtain the number of recombination events *r* per sequence pair. If *r* was an integer, then each sequence pair would undergo exactly *r* crossing over events. If *r* was a decimal number and *r* <1, then we introduced a single crossing over event into the pair with probability *r,* and no such event with probability 1-*r*. If *r* was a decimal number and *r* > 1, then *r* lay in the interval (*k*,*k* + 1), where *k* is some integer. In this case, we introduced *k* + 1 crossing over events with probability *r-k,* and *k* crossing over events with probability 1-(*r-k)*. Overall, our recombination procedure ensures that the number of recombination events is approximately the same as expected for a set of sequences with comparable diversity as that observed in our data.

In addition to the parameters described above, we constructed randomized network with higher recombination rates, to account for heterogeneous recombination rates across the genome, or higher effective population size, to account for higher effective population size for some genes such as HLA genes. Specifically, we constructed randomized networks with a ten-fold higher effective population size, i.e. 100,000 individuals, and randomized networks with twice the recombination rate that we had used initially. The new recombination rate is 1.90 cM/Mb.

The changes in recombination rate and effective population size did not change the final results. All the genes that were tested had more cycles in the giant component of their networks than any of the 1,000 randomized networks. Additional file 18: Figure S14 shows the mean and range of cycle count in the new randomized networks compared with the cycle count in original networks of the genes.

If many synonymous mutations are shared among sequences, the procedure from Eq. 1 would overestimate the number of needed recombination events if we simply counted the number *S* of synonymous changes across edges of a haplotype network. To find out whether this could be the case, we computed the number of synonymous changes that are shared among edges. (We note that each node in a haplotype network can correspond to multiple sequences that encode the same amino acid sequence, but may differ in synonymous changes, such that each edge can have multiple sets of associated synonymous changes.) To this end, we counted the fraction of synonymous changes on each edge that are also present in some other edge of the network. This fraction is small, with a median of 0.0459 and a mean of 0.0613. Thus, shared ancestry of synonymous changes is unlikely to confound our estimation of the number of recombination events.

#### XP-CLR neutrality test

We chose to use the XP-CLR (cross-population composite likelihood ratio) test [55] to test for neutral sequence evolution, because this test is robust to demographic history and recombination rate heterogeneity, and it detects both recent and ancient selective sweeps [55]. Briefly, the test searches for regions in the genome in which allele frequencies have changed too quickly to be explained by genetic drift. We used test statistics calculated for 2 kbp sliding windows calculated by [56] for the whole genome, based on the 1,000 genomes data [10]. Specifically, we performed this test for three populations, namely the CEU population (Utah Residents with Northern and Western European ancestry), the CHB population (Han Chinese in Beijing, China) and the YRI population (Yoruba in Ibadan, Nigeria) [75], which amounts to six possible population pairs and thus six calculations of the test statistics. To find the significance of the test statistics for any one gene of interest, we rank-ordered all the 2 kb windows in the genome by *p*-value, omitting windows with a value of the statistic equal to zero, i.e., lacking information. To identify candidate genes subject to positive selection, we determined which windows overlapped with each one of the 19,221 human genes. Only about 3 % of the windows that overlapped genes had a value of the statistic that indicated positive selection (at *p* = 0.05), but these windows overlapped with nearly 20 % of genes. This suggests that using this criterion to identify genes subject to positive selection would lead to a high false-discovery rate of positively selected genes. Therefore, we chose a more conservative criterion of calling only those genes subject to positive selection where at least two contiguous windows showed a significantly high test statistic (*p* = 0.01). According to this criterion, only 2 % of genes were subject to positive selection in each of the six population pairs.

### Calculating heterozygosity

To calculate the heterozygosity of any one gene, we used not haplotypes but (diploid) genotypes, and calculated the fraction of heterozygote individuals in our data set at each site where a non-synonymous amino acid change had occurred. We used the average of this value over all sites as our measure of the gene’s heterozygosity.

### Gene enrichment analysis

We used the g: Profiler web tool (Version: r1622_e84_eg31) [77] to ask if any gene ontology (GO) categories of biological processes and molecular functions or any pathways are significantly enriched in the 42 genes with a significant excess of squares in their haplotype network. In this analysis, we used default parameters of the tool, with two exceptions. First, we only searched for enrichment among GO biological processes and molecular functions, as well as among KEGG and Reactome pathways. Second, we set the hierarchical filtering of results, which provides a compact data representation, to “best per parent (moderate)”. GO terms are hierarchically related, and not filtering them hierarchically leads to unmanageably long and indiscriminate lists of enriched functions. The filtering uses the parent-wise grouping of significant terms and results in shorter GO output that is easier to analyze. Details of test results and parameters can be found in the electronic supplementary material.

### Gene conversion analysis

We used the GENECONV software on Linux (version 1.81a) [48] to detect gene conversion (with default parameters). The sequences that we supplied to the program included the haplotypes that comprised the giant component of the gene and included both synonymous and nonsynonymous changes.

## Abbreviations

ADGRV1 (ENSG00000164199), adhesion G protein-coupled receptor V1; AHNAK2 (ENSG00000185567), AHNAK nucleoprotein 2; ALPK2 (ENSG00000198796), alpha-kinase 2; CYP2A7 (ENSG00000198077), cytochrome P450, family 2, subfamily A, polypeptide 7; DCHS2 (ENSG00000197410), dachsous cadherin-related 2; DNAH11 (ENSG00000105877), dynein, axonemal, heavy chain 11; DNAH17 (ENSG00000187775), dynein, axonemal, heavy chain 17; DNAH5 (ENSG00000039139), dynein, axonemal, heavy chain 5; EYS (ENSG00000188107), eyes shut homolog (Drosophila); FBN3 (ENSG00000142449), fibrillin 3; FCGBP (ENSG00000090920), Fc fragment of IgG binding protein; FLG (ENSG00000143631), filaggrin; FRAS1 (ENSG00000138759), Fraser extracellular matrix complex subunit 1; HLA-A (ENSG00000206503), major histocompatibility complex, class I, A; HLA-B (ENSG00000234745), major histocompatibility complex, class I, B; HLA-C (ENSG00000204525), major histocompatibility complex, class I, C; HLA-DPB1 (ENSG00000223865), major histocompatibility complex, class II, DP beta 1; HLA-DRB1 (ENSG00000196126), major histocompatibility complex, class II, DR beta 1; HRNR (ENSG00000197915), hornerin; IGFN1 (ENSG00000163395), immunoglobulin-like and fibronectin type III domain containing 1; LAMA5 (ENSG00000130702), laminin, alpha 5; LILRA6 (ENSG00000244482), leukocyte immunoglobulin-like receptor, subfamily A (with TM domain), member 6; LILRB3 (ENSG00000204577), leukocyte immunoglobulin-like receptor, subfamily B (with TM and ITIM domains), member 3; MKI67 (ENSG00000148773), marker of proliferation Ki-67; MUC12 (ENSG00000205277), mucin 12, cell surface associated; MUC16 (ENSG00000181143), mucin 16, cell surface associated; MUC17 (ENSG00000169876), mucin 17, cell surface associated; MUC4 (ENSG00000145113), mucin 4, cell surface associated; MUC5B (ENSG00000117983), mucin 5B, oligomeric mucus/gel-forming; NEB (ENSG00000183091), nebulin; OBSCN (ENSG00000154358), obscurin, cytoskeletal calmodulin and titin-interacting RhoGEF; PCLO (ENSG00000186472), piccolo presynaptic cytomatrix protein; PKD1L1 (ENSG00000158683), polycystic kidney disease 1 like 1; PKD1L2 (ENSG00000166473), polycystic kidney disease 1-like 2 (gene/pseudogene); PLIN4 (ENSG00000167676), perilipin 4; POTED (ENSG00000166351), POTE ankyrin domain family, member D; PRAMEF2 (ENSG00000120952), PRAME family member 2; PRUNE2 (ENSG00000106772), prune homolog 2 (Drosophila); SYNE2 (ENSG00000054654), spectrin repeat containing, nuclear envelope 2; TG (ENSG00000042832), thyroglobulin; TTN (ENSG00000155657), itin; USH2A (ENSG00000042781), Usher syndrome 2A; XP-CLR, Cross-population composite likelihood ratio test

## Additional files

Additional file 1: Figure S1. Illustration of two haplotype networks, one highly connected and the other highly fragmented. a) Haplotype network of gene OTOG (Otogelin). Among all protein-based haplotype networks comprising more than 100 sequences, OTOG has the network with the largest giant component where all nodes fall into this component (181 nodes and a single component). b) Haplotype network of gene HLA-B, which is the most fragmented network, with 1,545 nodes in 1,111 components. Circles in a) and b) correspond to different genotypes, while edges connect genotypes that differ by a single point mutation. Circle color corresponds to the degree (number of neighbors) of the node, where darker nodes have a higher degree, and circle size corresponds to the number of haploid individuals with that genotype, where larger nodes are shared among more haploid individuals (PDF 3358 kb) Additional file 2: Figure S2. Cycles in haplotype networks illustrated with the example of a hexagon and an octagon. Circles (nodes) correspond to genotypes. An edge connects two nodes if they differ by a single mutation. Lettering next to each node indicates the nucleotides at which two genotypes differ. Edge labels show changes required to create a genotype from its neighbor, e.g., “A20G” indicates a change from A to G at position 20 of the hypothetical sequence. a) hypothetical hexagon in which six nucleotide changes occur, two each at positions 10, 20 and 30. If one starts from genotype 1, this genotype mutates twice and produces genotypes 2 and 3. Those genotypes in turn mutate to produce genotypes 4 and 5. Then either genotype 4 mutates at position 30 from A to T, or genotype 5 mutates at position 10 from A to G, or both of these mutations happen together, to produce genotype 6. This can be happen when there are evolutionary constraints that restrict other mutations. Recombination can also be responsible for this pattern. This pattern will be the same if one starts from any other node. b) hypothetical octagon in which eight nucleotide changes occur, two each at positions 10, 20, 30, and 40. Same pattern that was explained for a) can be explained here, with the only difference that there are more positions that are mutating. (PDF 193 kb) Additional file 3: This ZIP file contains the following documents. 1. genes_with_excess_of_cycles_exonLength_nucleotideMutations.txt: List of genes with excess of cycles when randomized networks are constructed from entire gene coding sequences (all four nucleotide mutations allowed). 2. gprofiler_results_100fragmentedGenes_largerthan10.xlsx: Gene ontology enrichment analysis results for the 100 most fragmented genes with a network comprising more than 10 sequences. 3. gprofiler_results_GO_function_process.xlsx: Gene ontology enrichment analysis of molecular functions and biological processes for the 42 genes with an excess of cycles. 4. gprofiler_results_GO_function_process_NoHLAGenes.xlsx: Gene ontology enrichment analysis of molecular functions and biological processes for the 42 genes with an excess of cycles, excluding the HLA genes. 5. gprofiler_results_KEGG.xlsx: KEGG pathways enrichment analysis for the 42 genes with an excess of cycles. 6. gprofiler_results_reactome.xlsx: Reactome pathways enrichment analysis for the 42 genes with an excess of cycles. 7. list_of_most_fragmented_networks.txt: List of 100 most fragmented networks among all haplotype networks comprising more than 10 nodes. Fragmentation is computed as the number of components divided by the size of the network (higher values correspond to greater fragmentation). (ZIP 63 kb) Additional file 4: Figure S3. Frequency of squares, hexagons and octagons among the 42 genes with an excess of cycles. The plot shows the frequency of elementary cycles of length 4, 6 and 8 in the giant component of genes with an excess of squares in their haplotype network. Note that the apparent discrepancy to Fig. 3a comes from the fact that Fig. 3a shows cycle numbers for haplotype networks of all genes. (PDF 296 kb) Additional file 5: Figure S4. Distribution of the size of the largest component in haplotype networks of 42 genes with an excess of squares in the largest component. The smallest giant component occurs in the network of *MKI67* (marker of proliferation Ki-67) with only 23 nodes, and the largest one occurs in the network of *DNAH11* (dynein, axonemal, heavy chain 11) with 538 nodes. (PDF 177 kb) Additional file 6: Figure S5. Distribution of the number of components in haplotype networks of 42 genes with an excess of squares in their largest component. The number of components ranges from one for gene *POTED* (POTE ankyrin domain family, member D) to 1,111 for the highly fragmented network of *HLA-B*. (PDF 63 kb) Additional file 7: Figure S6. Two examples for the distribution of the number of neighbors in the giant component of networks with an excess of squares. Most haplotype networks have a skewed distribution of the number of neighbors, of which the distribution in a) for *PKD1L1* (polycystic kidney disease 1 like 1) is representative. A minority of haplotype networks have a more symmetric distribution of this number of neighbors, as exemplified by b) for the network of *PRAMEF2* (PRAME family member 2). (PDF 135 kb) Additional file 8: Figure S7. Assortativity coefficient of haplotype networks of genes with an excess of squares. A graph is (dis)assortative if nodes with many neighbors tend to connect with other nodes that have many (few) neighbors. This property can be quantified through an assortativity coefficient, which is the Pearson correlation coefficient of degrees between every pair of neighboring nodes [83]. The higher this assortativity coefficient, the higher the tendency of a node to connect to other nodes with similar number of neighbors. The graph shows the assortativity coefficient (vertical axis) for the largest component of the haplotype network of each gene with a significant excess of squares (horizontal axis). (PDF 259 kb) Additional file 9: Figure S8. Recombination cannot produce the observed number of squares. For each of 41 genes with a significant excess of squares (horizontal axis), the vertical axis shows the number of squares in the largest components of the gene’s haplotype network (black circles), and the mean number of squares for corresponding networks created through 1,000 population simulations with recombination (blue circles, see Methods). The shaded area shows the minimum and maximum number of squares in 1,000 randomized networks for each gene. From the 42 genes with an excess of cycles, one gene (POTED, i.e., POTE ANKYRIN DOMAIN FAMILY, MEMBER D) was excluded from the analysis because it did not have any synonymous mutations, and so we could not estimate its recombination rate. (PDF 635 kb) Additional file 10: Table S1. Genes that showed a signal of positive selection in the XP-CLR (cross-population composite likelihood ratio) test [55]. Column one shows gene names and column two show the populationpairs in which the gene was identified as significant. Numbers in front of population pairs show the p-value of the most significant test statistic window overlapping the gene. CEU: Utah Residents with Northern and Western European ancestry; CHB: Han Chinese in Beijing, China;YRI: Yoruba in Ibadan Nigeria. (DOC 29 kb) Additional file 11: Table S2. Genes under positive selection as detected from Selectome database [54]. The Selectome database computes d_N_ / d_S_ ratio on branches of the phylogenetic tree of vertebrates and, after correcting for multiple testing, identifies genes that have a d_N_ / d_S_ ratio exceeding one on any specific tree branch. The table shows genes among the 42 genes with excess of squares in their network’s giant component that were detected by Selectome to be under positive selection. The second column shows that branch on which the gene was detected to be under positive selection. (DOC 32 kb) Additional file 12: Table S3. Hypergeometric test on genes under positive selection according to the XP-CLR test. From a total of six possible tests between pairs of genes in the three populations (YRI, CEU and CHB), only four tests showed evidence that any of the 42 genes with an excess of cycles were under positive selection. Table columns, from left to right, show the corresponding population pairs, the total number of genes in the analysis, the number of genes under positive selection according to the test, the number of genes under positive selection among the 42 genes with an excess of cycles, and the *p*-value of the hypergeometric test. A *p*-value lower than 0.01 indicates that it is unlikely to find as many genes in our dataset to be under positive selection by chance alone. (DOC 29 kb) Additional file 13: Figure S9. Fraction of edges without a single synonymous change (horizontal axis) in the giant component and the whole haplotype network of those 42 genes (left vertical axis) with significantly more squares than expected by chance alone. The numbers on the right vertical axis show the size of each haplotype network. (PDF 219 kb) Additional file 14: Figure S10. The fraction of edges without a single synonymous change inside and outside squares. For each of 42 genes (horizontal axis) with significantly more squares than expected by chance alone, vertical bars show the fraction of edges with no synonymous change for edges that are part of a square (black bars) and that are not part of a square (red bars). The fraction of edges without synonymous mutations is not significantly different for edges inside squares compared to edges outside squares for any gene (Mann-Whitney U test at *p* = 0.05 – corrected for multiple testing using [46]). (PDF 224 kb) Additional file 15: Figure S11. Association between gene heterozygosity and number of squares in the giant component of a gene’s haplotype network. We calculated the heterozygosity of each gene (*n* = 12,235) as the average fraction of individuals heterozygous in that gene, where we took the average across all polymorphic sites in the population. The correlation is very weak but significant (Pearson’s *r* = 0.066; *p* = 3.42 × 10^-13^; *n* = 12,235). The blue line is based on linear regression. (PDF 162 kb) Additional file 16 : Figure S12. Correlation between the size of the giant component and the number of cycles in the giant component of haplotype networks (based on 12235 genes). Lines show results of linear regression analysis. Red specifies genes with a significant excess of cycles in their giant component (42 genes). The size of the giant component and the number of cycles are significantly correlated both across all genes and across genes with an excess of cycles (Pearson’s product-moment correlation, *p*-value = 2.2 × 10^-16^ and *p*-value = 1.04 × 10^-13^ for all genes and for genes with an excess of cycles). (PDF 600 kb) Additional file 17: Figure S13. Distribution of the size of the giant component in gene haplotype networks. The left panel shows this distribution for all 12,235 genes with at least one amino acid changing mutation (mean size of 35.7 haplotypes), and the right panel shows the distribution for those 42 genes with excess of cycles (mean size of 179.0 haplotypes). The two distributions are significantly different from each other (independent 2-group Mann- Whitney U Test, *p*-value = 2.2 × 10–16). (PDF 478 kb) Additional file 18: Figure S14. Elevated recombination rates or increased effective population size cannot explain the observed number of cycles. The vertical axes show the number of squares in the largest components of a gene’s haplotype network (black circles), and the mean number of squares for corresponding networks created through 1,000 population simulations with recombination (blue circles, see Methods). The shaded areas show the minimum and maximum number of squares in 1,000 randomized networks for each gene. a) Randomized networks were constructed with twice the average recombination rate than in the human genes, i.e. 1.90 cM/Mb. b) Randomized networks were constructed based on ten times the estimated effective population size of humans, i.e. 100,000 individuals. All other calculations and procedures are the same as described in the Methods section describing how randomized networks with recombination were generated. From the 42 genes with an excess of cycles, one gene (POTED) was excluded from the analysis because it did not have any synonymous mutations, and so we could not estimate its recombination rate. (PDF 1358 kb)
