# Supplementary figures and images for: Parallel or convergent evolution in human population genomic data revealed by genotype networks
Source: BMC Evol Biol. 2016 Aug 2;16:154. doi: 10.1186/s12862-016-0722-0 (PMC4969671; doi:10.1186/s12862-016-0722-0)

a) OTOG

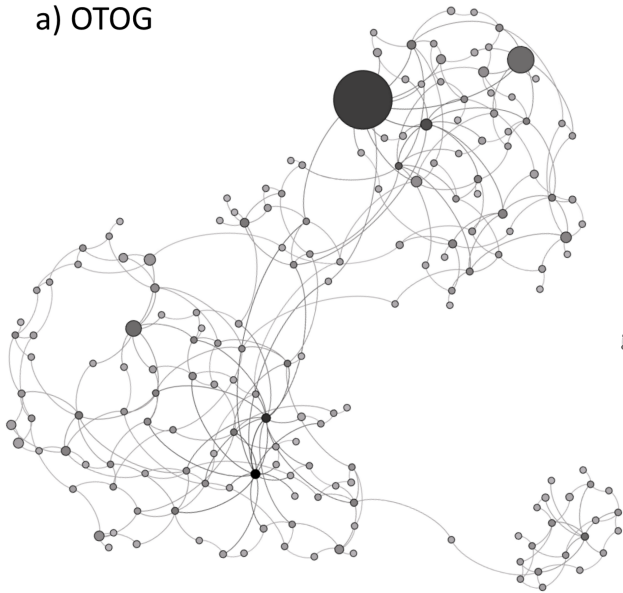

b) HLA-B

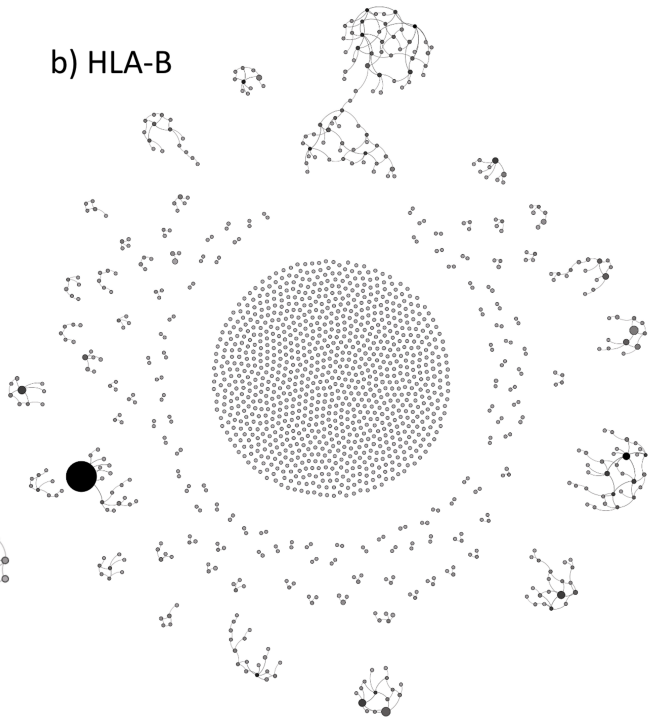

Supplement: Additional file 1: Figure S1. — Illustration of two haplotype networks, one highly connected and the other highly fragmented. a) Haplotype network of gene OTOG (Otogelin). Among all protein-based haplotype networks comprising more than 100 sequences, OTOG has the network with the largest giant component where all nodes fall into this component (181 nodes and a single component). b) Haplotype network of gene HLA-B, which is the most fragmented network, with 1,545 nodes in 1,111 components. Circles in a) and b) correspond to different genotypes, while edges connect genotypes that differ by a single point mutation. Circle color corresponds to the degree (number of neighbors) of the node, where darker nodes have a higher degree, and circle size corresponds to the number of haploid individuals with that genotype, where larger nodes are shared among more haploid individuals (PDF 3358 kb) [file 12862_2016_722_MOESM1_ESM.pdf]

a)

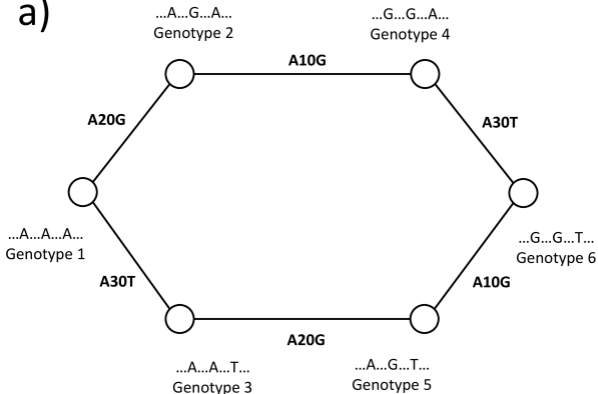

b)

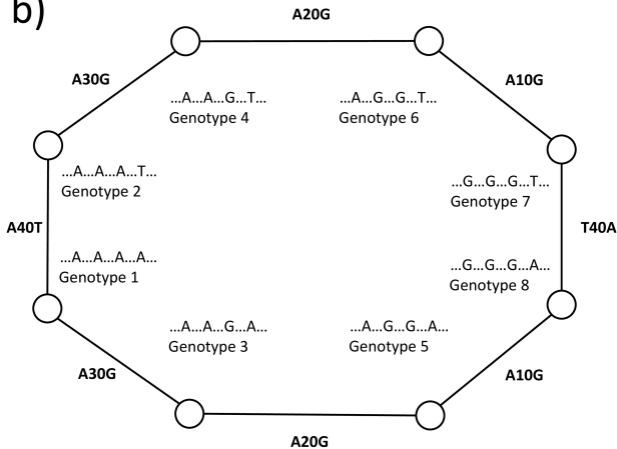

Supplement: Additional file 2: Figure S2. — Cycles in haplotype networks illustrated with the example of a hexagon and an octagon. Circles (nodes) correspond to genotypes. An edge connects two nodes if they differ by a single mutation. Lettering next to each node indicates the nucleotides at which two genotypes differ. Edge labels show changes required to create a genotype from its neighbor, e.g., “A20G” indicates a change from A to G at position 20 of the hypothetical sequence. a) hypothetical hexagon in which six nucleotide changes occur, two each at positions 10, 20 and 30. If one starts from genotype 1, this genotype mutates twice and produces genotypes 2 and 3. Those genotypes in turn mutate to produce genotypes 4 and 5. Then either genotype 4 mutates at position 30 from A to T, or genotype 5 mutates at position 10 from A to G, or both of these mutations happen together, to produce genotype 6. This can be happen when there are evolutionary constraints that restrict other mutations. Recombination can also be responsible for this pattern. This pattern will be the same if one starts from any other node. b) hypothetical octagon in which eight nucleotide changes occur, two each at positions 10, 20, 30, and 40. Same pattern that was explained for a) can be explained here, with the only difference that there are more positions that are mutating. (PDF 193 kb) [file 12862_2016_722_MOESM2_ESM.pdf]

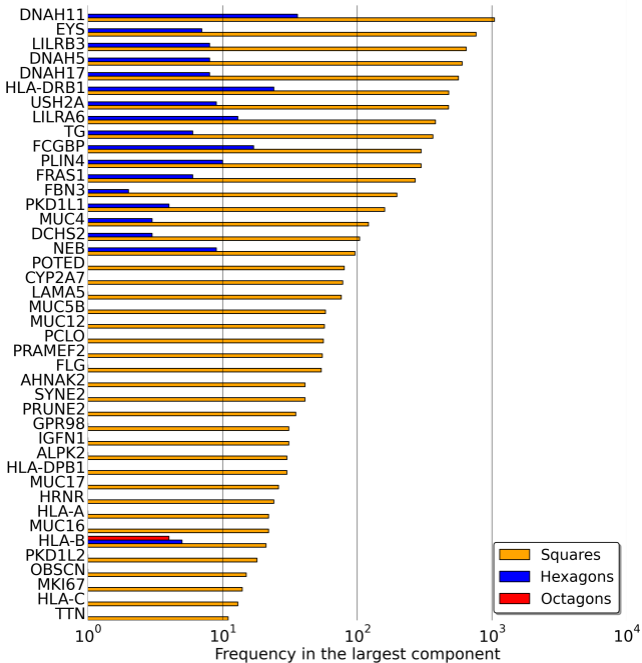

Supplement: Additional file 4: Figure S3. — Frequency of squares, hexagons and octagons among the 42 genes with an excess of cycles. The plot shows the frequency of elementary cycles of length 4, 6 and 8 in the giant component of genes with an excess of squares in their haplotype network. Note that the apparent discrepancy to Fig. 3a comes from the fact that Fig. 3a shows cycle numbers for haplotype networks of all genes. (PDF 296 kb) [file 12862_2016_722_MOESM4_ESM.pdf]

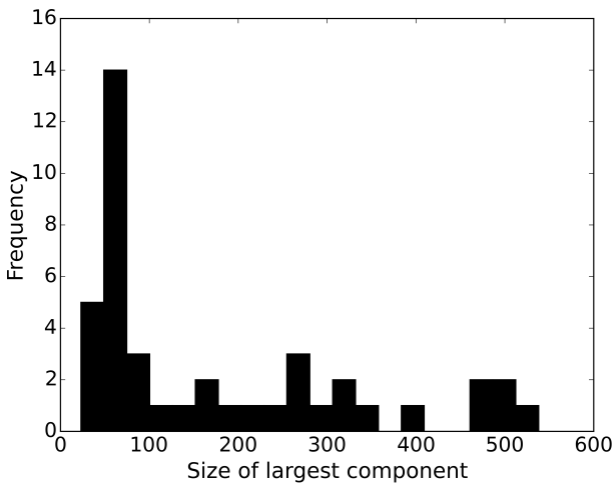

Supplement: Additional file 5: Figure S4. — Distribution of the size of the largest component in haplotype networks of 42 genes with an excess of squares in the largest component. The smallest giant component occurs in the network of MKI67 (marker of proliferation Ki-67) with only 23 nodes, and the largest one occurs in the network of DNAH11 (dynein, axonemal, heavy chain 11) with 538 nodes. (PDF 177 kb) [file 12862_2016_722_MOESM5_ESM.pdf]

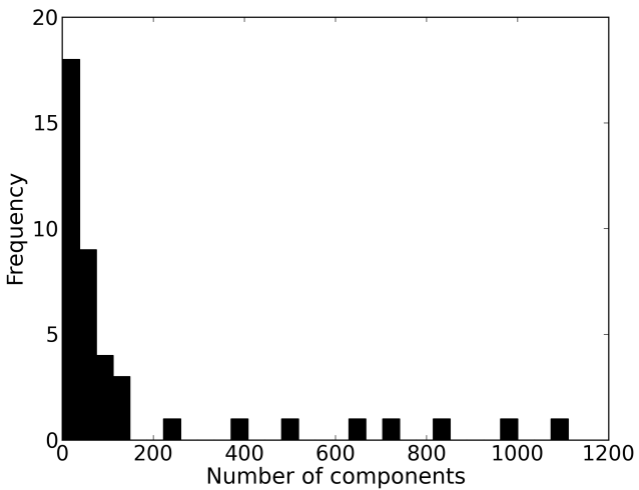

Supplement: Additional file 6: Figure S5. — Distribution of the number of components in haplotype networks of 42 genes with an excess of squares in their largest component. The number of components ranges from one for gene POTED (POTE ankyrin domain family, member D) to 1,111 for the highly fragmented network of HLA-B. (PDF 63 kb) [file 12862_2016_722_MOESM6_ESM.pdf]

a) PKD1L1

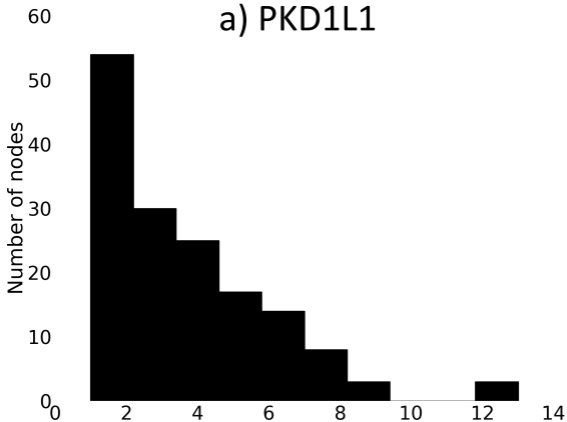

b) PRAMEF2

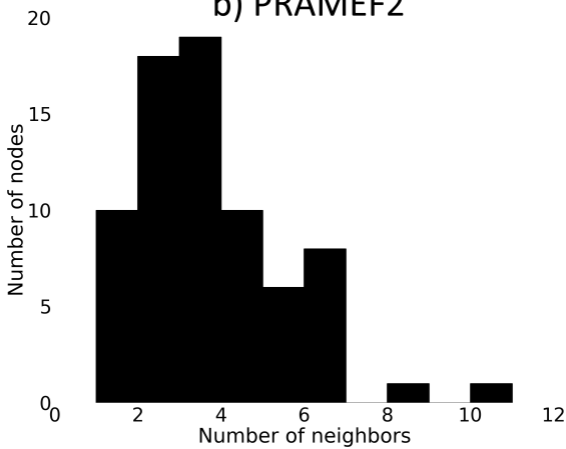

Supplement: Additional file 7: Figure S6. — Two examples for the distribution of the number of neighbors in the giant component of networks with an excess of squares. Most haplotype networks have a skewed distribution of the number of neighbors, of which the distribution in a) for PKD1L1 (polycystic kidney disease 1 like 1) is representative. A minority of haplotype networks have a more symmetric distribution of this number of neighbors, as exemplified by b) for the network of PRAMEF2 (PRAME family member 2). (PDF 135 kb) [file 12862_2016_722_MOESM7_ESM.pdf]

Assortativity coefficient  
of the giant component

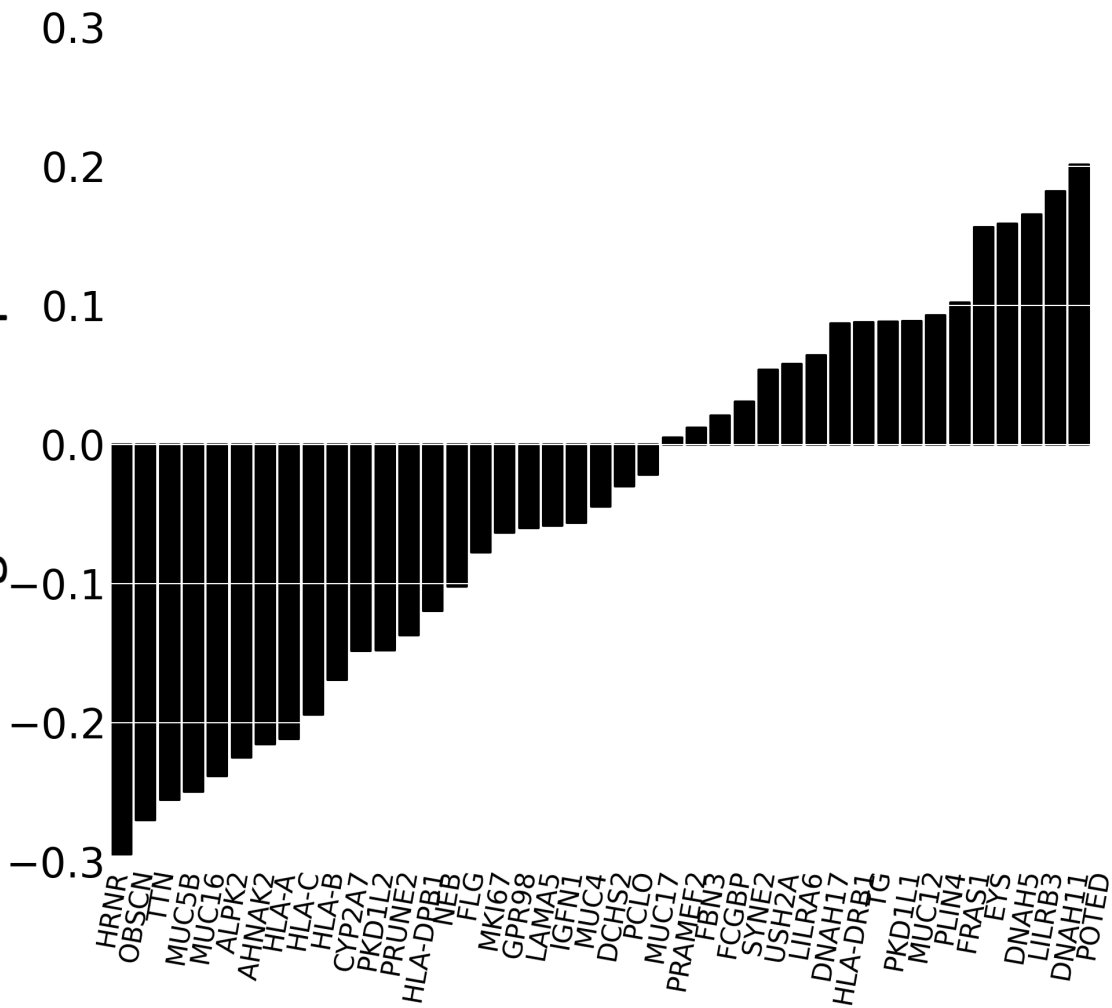

Supplement: Additional file 8: Figure S7. — Assortativity coefficient of haplotype networks of genes with an excess of squares. A graph is (dis)assortative if nodes with many neighbors tend to connect with other nodes that have many (few) neighbors. This property can be quantified through an assortativity coefficient, which is the Pearson correlation coefficient of degrees between every pair of neighboring nodes [83]. The higher this assortativity coefficient, the higher the tendency of a node to connect to other nodes with similar number of neighbors. The graph shows the assortativity coefficient (vertical axis) for the largest component of the haplotype network of each gene with a significant excess of squares (horizontal axis). (PDF 259 kb) [file 12862_2016_722_MOESM8_ESM.pdf]

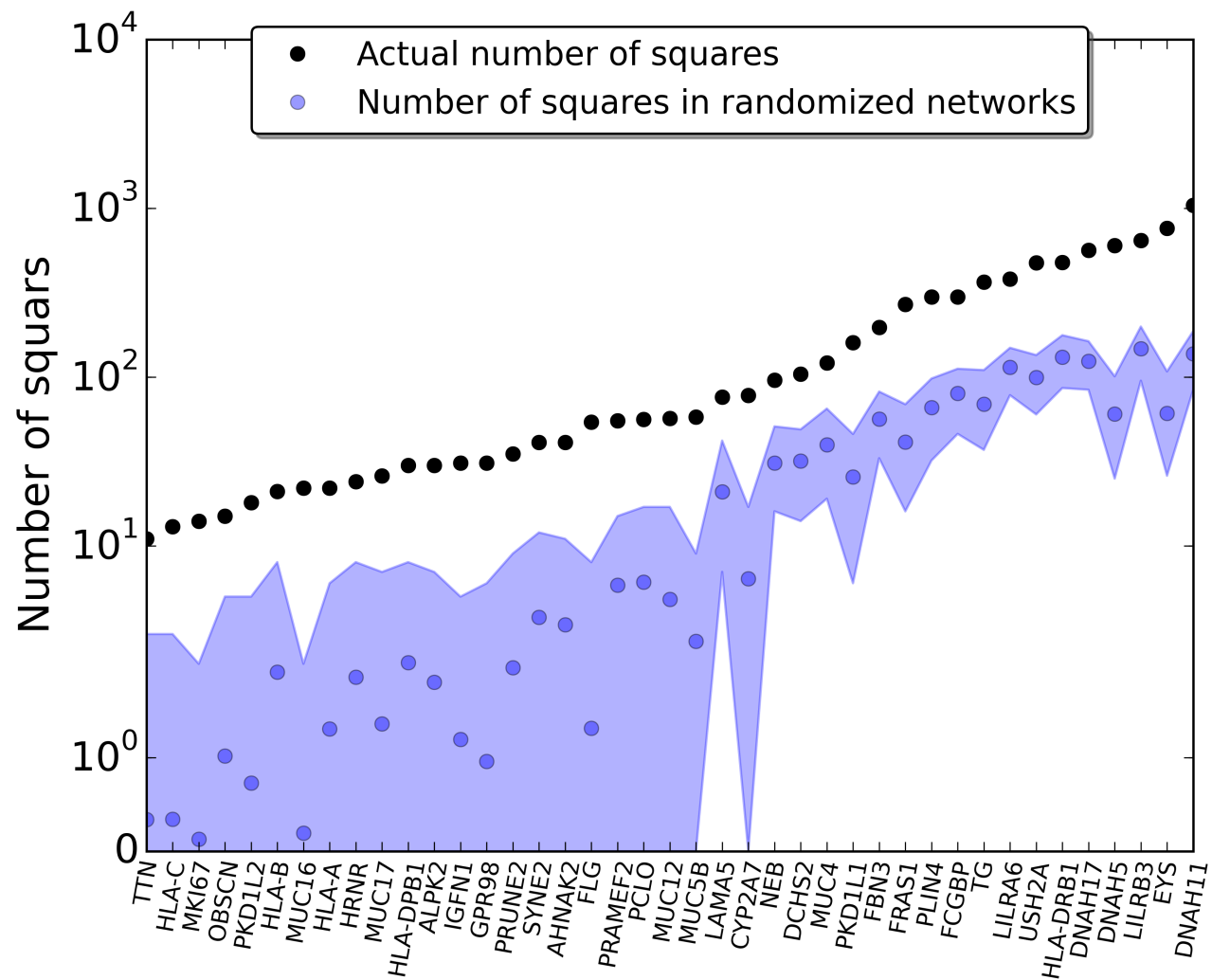

Supplement: Additional file 9: Figure S8. — Recombination cannot produce the observed number of squares. For each of 41 genes with a significant excess of squares (horizontal axis), the vertical axis shows the number of squares in the largest components of the gene’s haplotype network (black circles), and the mean number of squares for corresponding networks created through 1,000 population simulations with recombination (blue circles, see Methods). The shaded area shows the minimum and maximum number of squares in 1,000 randomized networks for each gene. From the 42 genes with an excess of cycles, one gene (POTED, i.e., POTE ANKYRIN DOMAIN FAMILY, MEMBER D) was excluded from the analysis because it did not have any synonymous mutations, and so we could not estimate its recombination rate. (PDF 635 kb) [file 12862_2016_722_MOESM9_ESM.pdf]

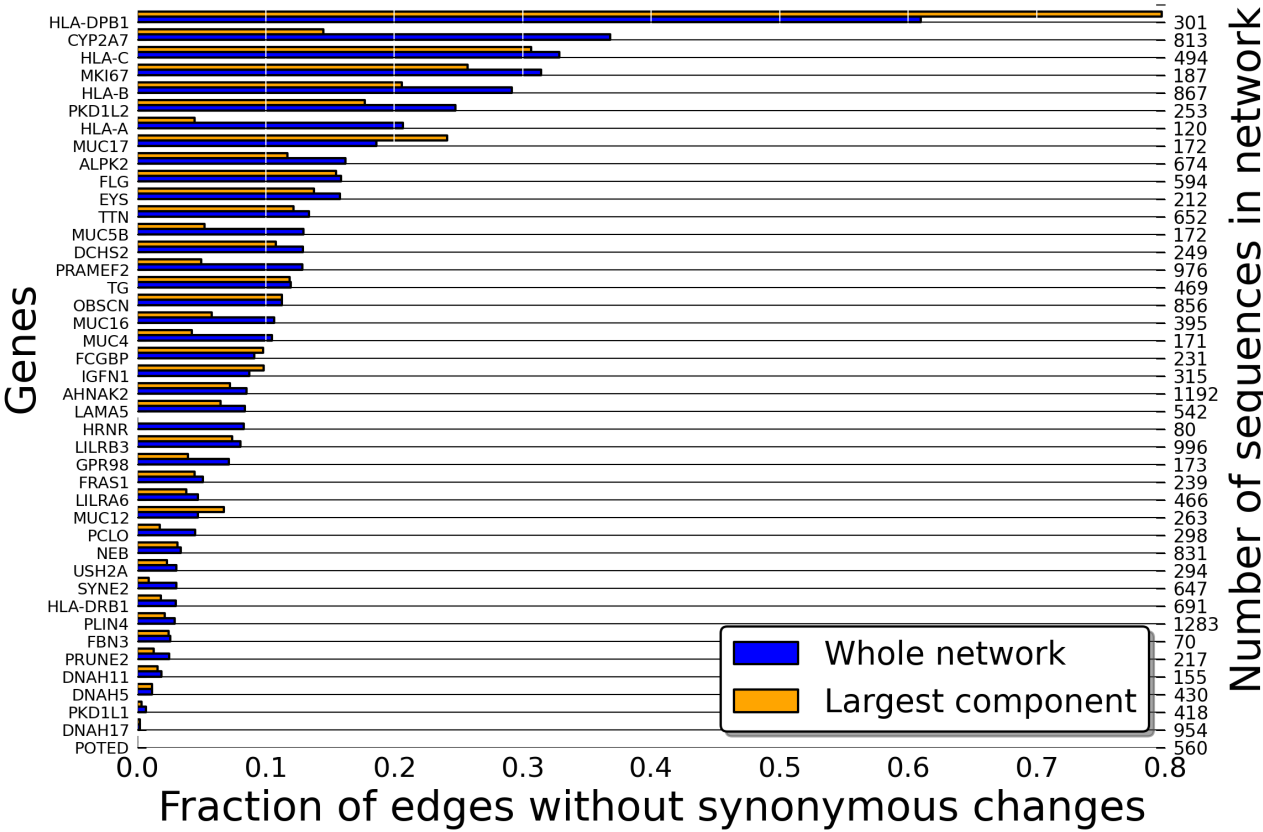

Supplement: Additional file 13: Figure S9. — Fraction of edges without a single synonymous change (horizontal axis) in the giant component and the whole haplotype network of those 42 genes (left vertical axis) with significantly more squares than expected by chance alone. The numbers on the right vertical axis show the size of each haplotype network. (PDF 219 kb) [file 12862_2016_722_MOESM13_ESM.pdf]

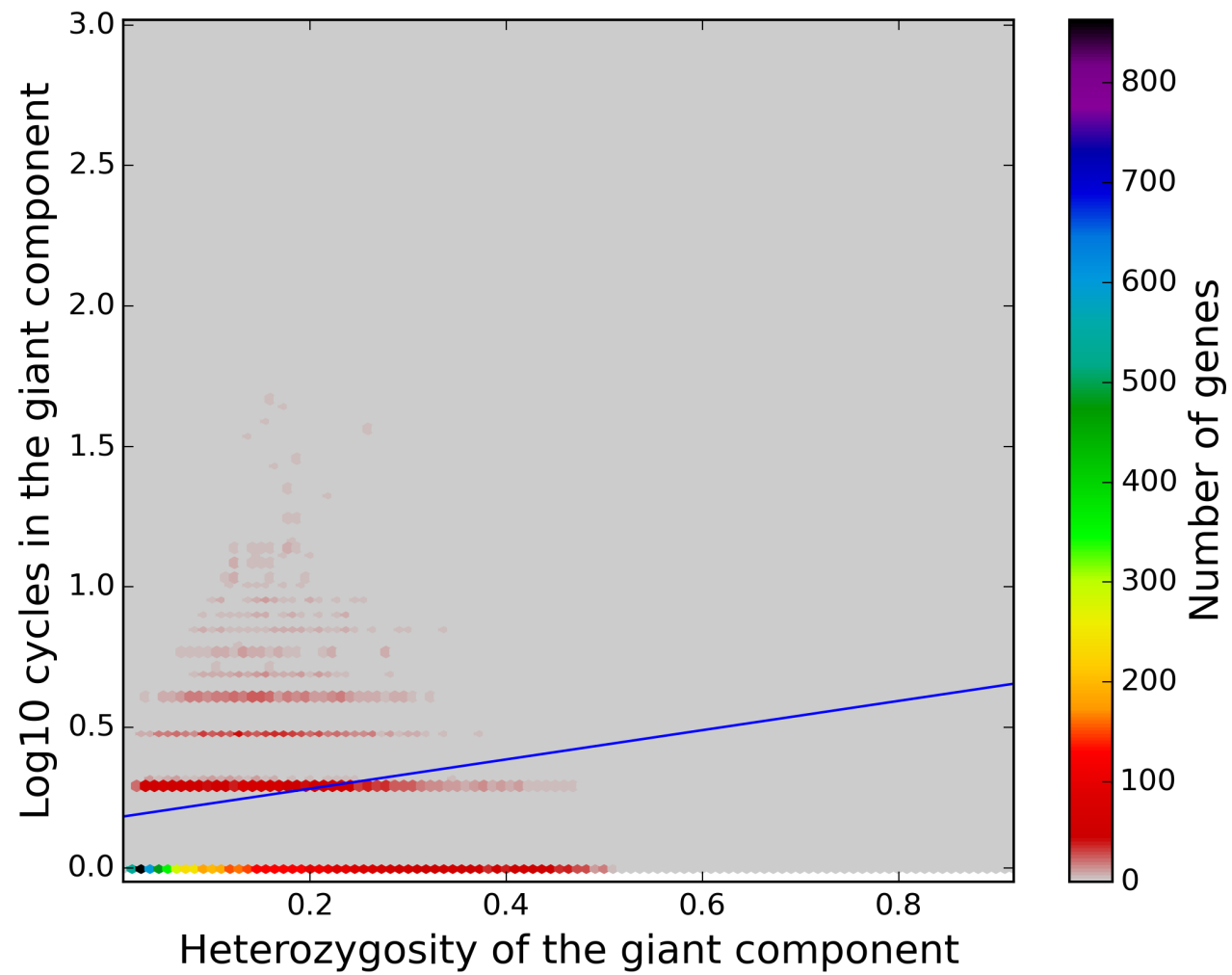

Supplement: Additional file 15: Figure S11. — Association between gene heterozygosity and number of squares in the giant component of a gene’s haplotype network. We calculated the heterozygosity of each gene (n = 12,235) as the average fraction of individuals heterozygous in that gene, where we took the average across all polymorphic sites in the population. The correlation is very weak but significant (Pearson’s r = 0.066; p = 3.42 × 10^-13^; n = 12,235). The blue line is based on linear regression. (PDF 162 kb) [file 12862_2016_722_MOESM15_ESM.pdf]

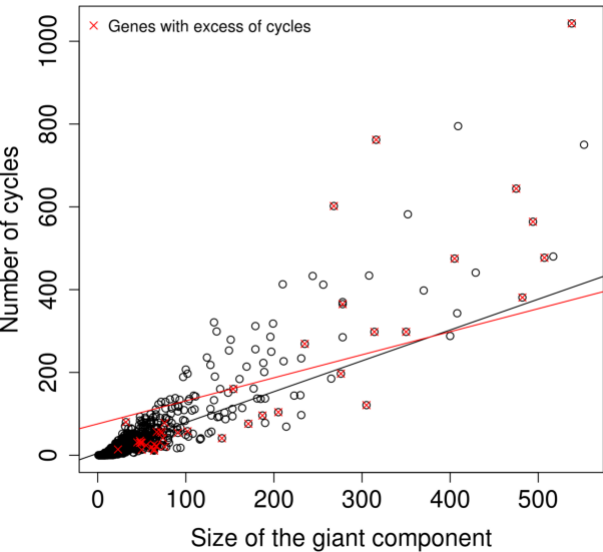

Supplement: Additional file 16 : Figure S12. — Correlation between the size of the giant component and the number of cycles in the giant component of haplotype networks (based on 12235 genes). Lines show results of linear regression analysis. Red specifies genes with a significant excess of cycles in their giant component (42 genes). The size of the giant component and the number of cycles are significantly correlated both across all genes and across genes with an excess of cycles (Pearson’s product-moment correlation, p-value = 2.2 × 10^-16^ and p-value = 1.04 × 10^-13^ for all genes and for genes with an excess of cycles). (PDF 600 kb) [file 12862_2016_722_MOESM16_ESM.pdf]

**All genes**

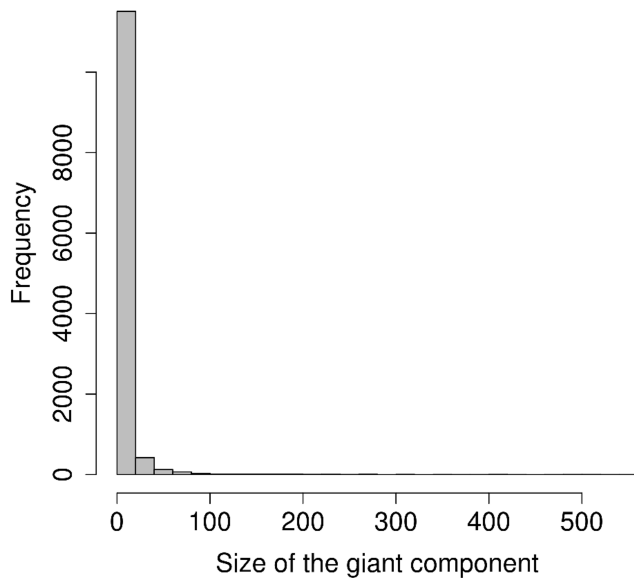

**Genes with excess of cycles**

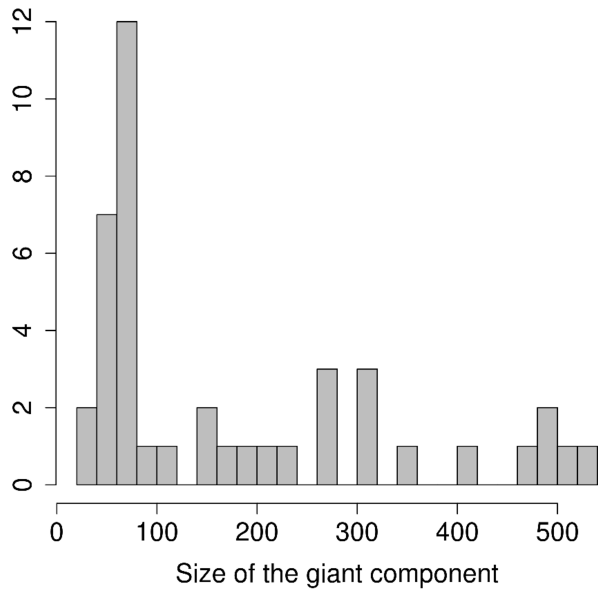

Supplement: Additional file 17: Figure S13. — Distribution of the size of the giant component in gene haplotype networks. The left panel shows this distribution for all 12,235 genes with at least one amino acid changing mutation (mean size of 35.7 haplotypes), and the right panel shows the distribution for those 42 genes with excess of cycles (mean size of 179.0 haplotypes). The two distributions are significantly different from each other (independent 2-group Mann- Whitney U Test, p-value = 2.2 × 10^–16^). (PDF 478 kb) [file 12862_2016_722_MOESM17_ESM.pdf]

Number of squares

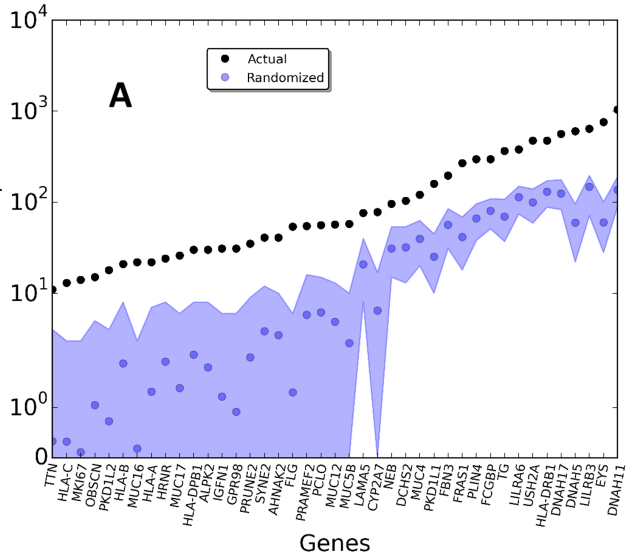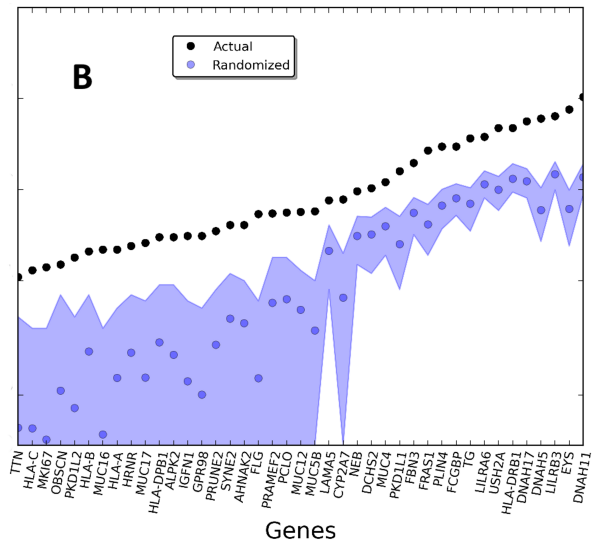

Supplement: Additional file 18: Figure S14. — Elevated recombination rates or increased effective population size cannot explain the observed number of cycles. The vertical axes show the number of squares in the largest components of a gene’s haplotype network (black circles), and the mean number of squares for corresponding networks created through 1,000 population simulations with recombination (blue circles, see Methods). The shaded areas show the minimum and maximum number of squares in 1,000 randomized networks for each gene. a) Randomized networks were constructed with twice the average recombination rate than in the human genes, i.e. 1.90 cM/Mb. b) Randomized networks were constructed based on ten times the estimated effective population size of humans, i.e. 100,000 individuals. All other calculations and procedures are the same as described in the Methods section describing how randomized networks with recombination were generated. From the 42 genes with an excess of cycles, one gene (POTED) was excluded from the analysis because it did not have any synonymous mutations, and so we could not estimate its recombination rate. (PDF 1358 kb) [file 12862_2016_722_MOESM18_ESM.pdf]
