# Supplementary material for: Parallel or convergent evolution in human population genomic data revealed by genotype networks
Source: BMC Evol Biol. 2016 Aug 2;16:154. doi: 10.1186/s12862-016-0722-0 (PMC4969671; doi:10.1186/s12862-016-0722-0)
Supplement: Additional file 10: Table S1. — Genes that showed a signal of positive selection in the XP-CLR (cross-population composite likelihood ratio) test [55]. Column one shows gene names and column two show the populationpairs in which the gene was identified as significant. Numbers in front of population pairs show the p-value of the most significant test statistic window overlapping the gene. CEU: Utah Residents with Northern and Western European ancestry; CHB: Han Chinese in Beijing, China;YRI: Yoruba in Ibadan Nigeria. (DOC 29 kb) [file 12862_2016_722_MOESM10_ESM.doc]

Table S1

| **Gene name** | **Population pair** |
| --- | --- |
| *NEB* | CEU compared against YRI |
| *IGFN1* | YRI compared against CEU |
| *FLG* | YRI compared against CEU |
| *PKD1L1* | YRI compared against CEU |
| *GPR98* | CEU compared against CHB |
| *FRAS1* | YRI compared against CHB |
