# Supplementary material for: Parallel or convergent evolution in human population genomic data revealed by genotype networks
Source: BMC Evol Biol. 2016 Aug 2;16:154. doi: 10.1186/s12862-016-0722-0 (PMC4969671; doi:10.1186/s12862-016-0722-0)
Supplement: Additional file 11: Table S2. — Genes under positive selection as detected from Selectome database [54]. The Selectome database computes dN / dS ratio on branches of the phylogenetic tree of vertebrates and, after correcting for multiple testing, identifies genes that have a dN / dS ratio exceeding one on any specific tree branch. The table shows genes among the 42 genes with excess of squares in their network’s giant component that were detected by Selectome to be under positive selection. The second column shows that branch on which the gene was detected to be under positive selection. (DOC 32 kb) [file 12862_2016_722_MOESM11_ESM.doc]

Table S2

| **Gene** | **Taxon** |
| --- | --- |
| *DNAH5* | Euteleostomi |
| *USH2A* | Euteleostomi |
| *PRAMEF2* | Primates |
| *LAMA5* | Euteleostomi |
| *FRAS1* | Euteleostomi |
| *FBN3* | Euteleostomi |
| *IGFN1* | Euteleostomi |
| *GPR98* | Euteleostomi |
| *PCLO* | Euteleostomi |
| *DNAH17* | Euteleostomi |
| *HLA-DRB1* | Primates |
| *FCGBP* | Euteleostomi |
