# Supplementary material for: Parallel or convergent evolution in human population genomic data revealed by genotype networks
Source: BMC Evol Biol. 2016 Aug 2;16:154. doi: 10.1186/s12862-016-0722-0 (PMC4969671; doi:10.1186/s12862-016-0722-0)
Supplement: Additional file 12: Table S3. — Hypergeometric test on genes under positive selection according to the XP-CLR test. From a total of six possible tests between pairs of genes in the three populations (YRI, CEU and CHB), only four tests showed evidence that any of the 42 genes with an excess of cycles were under positive selection. Table columns, from left to right, show the corresponding population pairs, the total number of genes in the analysis, the number of genes under positive selection according to the test, the number of genes under positive selection among the 42 genes with an excess of cycles, and the p-value of the hypergeometric test. A p-value lower than 0.01 indicates that it is unlikely to find as many genes in our dataset to be under positive selection by chance alone. (DOC 29 kb) [file 12862_2016_722_MOESM12_ESM.doc]

Table S3

| Population | Genes in the population | Genes under positive selection in the population | Genes under positive selection that are part of our dataset | Hypergeometric test p-value |
| --- | --- | --- | --- | --- |
| CEU – YRI | 19221 | 371 | 1 | 0.19 |
| YRI – CEU | 19221 | 408 | 3 | 0.01 |
| CEU – CHB | 19221 | 342 | 1 | 0.17 |
| YRI – CHB | 19221 | 375 | 1 | 0.20 |
