# Supplementary material for: Parallel or convergent evolution in human population genomic data revealed by genotype networks
Source: BMC Evol Biol. 2016 Aug 2;16:154. doi: 10.1186/s12862-016-0722-0 (PMC4969671; doi:10.1186/s12862-016-0722-0)
Supplement: Additional file 14: Figure S10. — The fraction of edges without a single synonymous change inside and outside squares. For each of 42 genes (horizontal axis) with significantly more squares than expected by chance alone, vertical bars show the fraction of edges with no synonymous change for edges that are part of a square (black bars) and that are not part of a square (red bars). The fraction of edges without synonymous mutations is not significantly different for edges inside squares compared to edges outside squares for any gene (Mann-Whitney U test at p = 0.05 – corrected for multiple testing using [46]). (PDF 224 kb) [file 12862_2016_722_MOESM14_ESM.pdf]

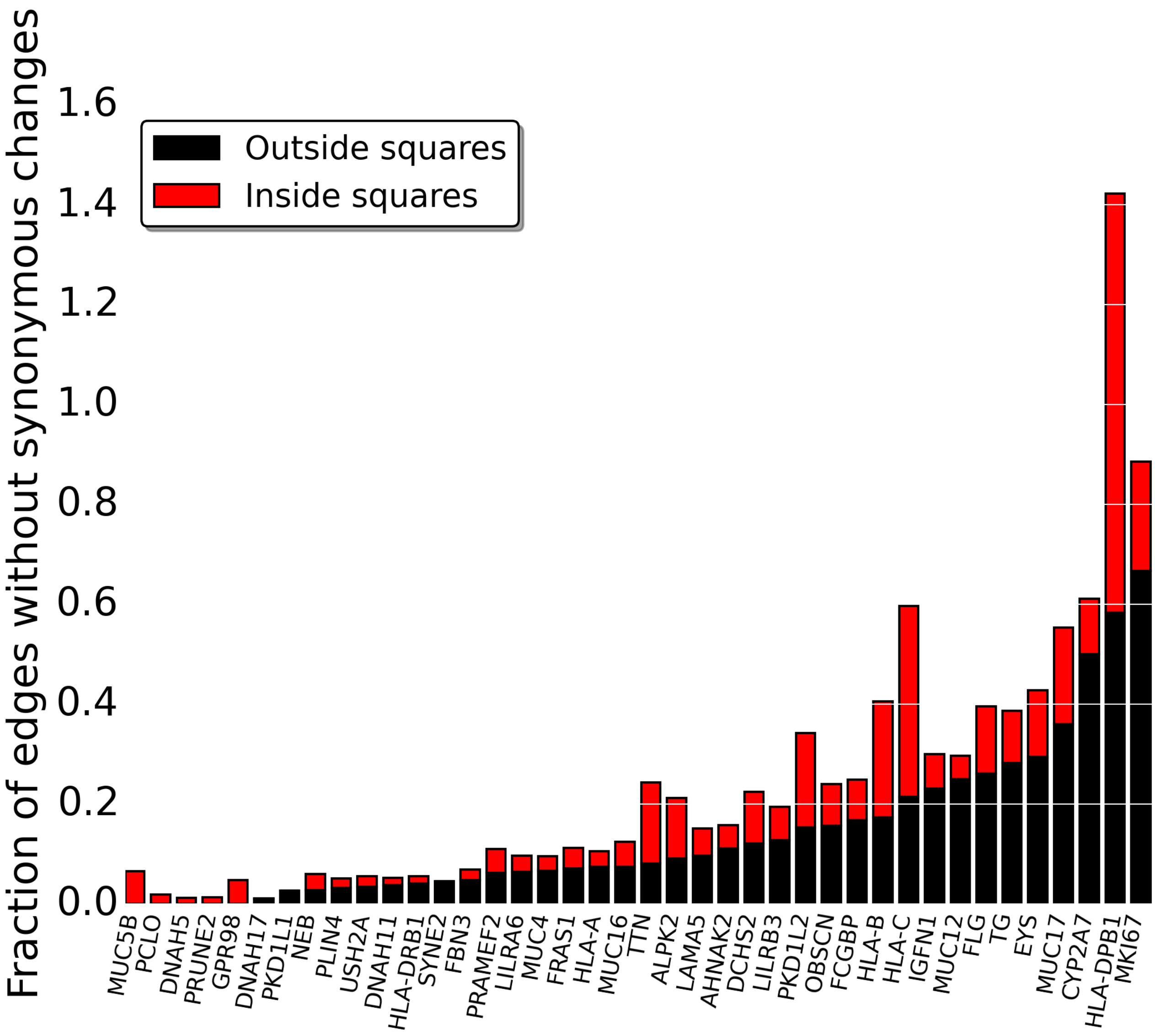

Fraction of edges without synonymous changes
0.0
0.2
0.4
0.6
0.8
1.0
1.2
1.4
1.6
Outside squares
Inside squares
MUC5B
PCLO
DNAH5
PRUNE2
GPR98
DNAH17
PKD1L1
NEB
PLIN4
USH2A
DNAH11
HLA-DRB1
SYNE2
FBN3
PRAMEF2
LILRA6
MUC4
FRAS1
HLA-A
MUC16
TTN
ALPK2
LAMA5
AHNAK2
DCHS2
LILRB3
PKD1L2
OBSCN
FCGBP
HLA-B
HLA-C
IGFN1
MUC12
FLG
TG
EYS
MUC17
CYP2A7
HLA-DPB1
MKI67
